# Genome-wide analysis, transcription factor network approach and gene expression profile of *GH3* genes over early somatic embryogenesis in *Coffea* spp

**DOI:** 10.1186/s12864-019-6176-1

**Published:** 2019-11-06

**Authors:** Renan Terassi Pinto, Natália Chagas Freitas, Wesley Pires Flausino Máximo, Thiago Bergamo Cardoso, Débora de Oliveira Prudente, Luciano Vilela Paiva

**Affiliations:** 1 Department of Chemistry, Federal University of Lavras, Lavras, MG 37200000 Brazil; 2 Sugarcane Technology Center, Piracicaba, SP 13400970 Brazil

**Keywords:** Gretchen Hagen 3, Auxin homeostasis, Phylogenetics, Baby Boom, Coffee clonal propagation

## Abstract

**Background:**

Coffee production relies on plantations with varieties from *Coffea arabica* and *Coffea canephora* species. The first, the most representative in terms of coffee consumption, is mostly propagated by seeds, which leads to management problems regarding the plantations maintenance, harvest and processing of grains. Therefore, an efficient clonal propagation process is required for this species cultivation, which is possible by reaching a scalable and cost-effective somatic embryogenesis protocol. A key process on somatic embryogenesis induction is the auxin homeostasis performed by Gretchen Hagen 3 (GH3) proteins through amino acid conjugation. In this study, the *GH3* family members were identified on *C. canephora* genome, and by performing analysis related to gene and protein structure and transcriptomic profile on embryogenic tissues, we point a *GH3* gene as a potential regulator of auxin homeostasis during early somatic embryogenesis in *C. arabica* plants.

**Results:**

We have searched within the published *C. canephora* genome and found 17 *GH3* family members. We checked the conserved domains for GH3 proteins and clustered the members in three main groups according to phylogenetic relationships. We identified amino acids sets in four GH3 proteins that are related to acidic amino acid conjugation to auxin, and using a transcription factor (TF) network approach followed by RT-qPCR we analyzed their possible transcriptional regulators and expression profiles in cells with contrasting embryogenic potential in *C. arabica*. The *CaGH3.15* expression pattern is the most correlated with embryogenic potential and with *CaBBM*, a *C. arabica* ortholog of a major somatic embryogenesis regulator.

**Conclusion:**

Therefore, one out of the *GH3* members may be influencing on coffee somatic embryogenesis by auxin conjugation with acidic amino acids, which leads to the phytohormone degradation. It is an indicative that this gene can serve as a molecular marker for coffee cells with embryogenic potential and needs to be further studied on how much determinant it is for this process. This work, together with future studies, can support the improvement of coffee clonal propagation through in vitro derived somatic embryos.

## Background

Coffee is a worldwide consumed commodity, mostly produced through *Coffea arabica* (63.21% of total production) and *Coffea canephora* plantations, with Brazil as the biggest producer country that corresponds to 35.7% of the global production [1]. The beverage generated from the roasted grains is mainly characterized by caffeine content and its effects as stimulant [2], but has other metabolite compounds, like flavonols, with antioxidant properties that are beneficial for human health [3].

The crop is predominantly propagated by seeds, which impair the plantation homogeneity. Rooting recalcitrance of plantlets is one of the main reasons by which common vegetative propagation has not been applied [4] yet, leading coffee researchers to the challenge of establishing an efficient alternative method for propagation. In vitro somatic embryogenesis (SE) followed by development and acclimatization of the plantlets is an interesting option for achieving efficient clonal propagation, as in 2016 around 7 million coffee plants were produced through this process in Central America [5].

Somatic embryogenesis is also an important process for genetic transformation, due to the possibility of regenerating plantlets from single cells or small cellular clusters, an alternative way to improve perennial crops breeding. One of the challenges is to understand what differentiates cells with embryogenic competence from the others, and possibly confirm or establish new molecular markers, as morphological characteristics alone are not enough to predict embryogenic capacity [6].

In the case of *C. arabica*, SE is achieved by the indirect pathway, that is, with an intermediate step of calli formation before embryo regeneration. The coffee leaf explants incubated on auxin-rich medium generate embryogenic-competent calli just after nearly three months, together with non-embryogenic callus production. This pattern of embryogenic calli formation seems to occur via root meristem-associated pathway, with the cellular identity being similar to root meristem cells, which is induced by incubation on auxin-rich medium and wounds, triggered mostly by auxin signaling and regulators such as ARFs and WOX11 [7].

Comprehension of metabolic pathways related to auxin homeostasis can be very informative because such hormone is among the major regulators of SE induction and embryo development [8, 9]. The balance between auxin and its conjugates with amino acids is determinant for cell responses to environment stimuli [10] and represents a deeper layer of complexity related to auxin balance influence on SE, as exemplified by the report that different conjugates are associated to specific direct somatic embryogenesis phases in *C. canephora* [9]. This conjugation between amino acids and auxin is catalyzed by Gretchen Hagen 3 (GH3) family proteins [10–12], which is a widespread family in plants [13] and have been recently characterized in some species like *Solanum lycopersicum* [14], *Malus domestica* [15] and *Medicago truncatula* [16].

Proteins of GH3 family catalyze amino acid conjugation to acyl substrates, mostly to auxin, jasmonic acid and benzoates, thus being associated to many plant metabolic pathways. It is reported that members of this family can be clustered into three main groups and, possibly, proteins from the same group share similarities regarding the specificity to acyl substrates [13]. Generally, specific sets of amino acid residues are related to protein interaction with specific substrates and they are different among GH3 proteins with different substrate affinity [11, 17]. Therefore, the knowledge about the relations of these specific amino acid sequences with substrate specificity is helpful in the search for a proper *GH3* gene related to a specific study subject.

According to this context, our aim was to identify the members of *GH3* family in *C. canephora*, the *Coffea* species with an available public genome, and analyze their phylogenetic and structural features, as well as transcriptional profile and point transcription factors related to potential SE regulators *GH3* members. We have found four potential members (CcGH3.9, CcGH3.13, CcGH3.15 and CcGH3.16) that may be associated with auxin conjugation to acidic amino acids which can lead auxin to degradation [17], and some of their potential transcriptional regulators. We analyzed the transcriptional profile of these four homologous *CcGH3s* genes in *C. arabica* calli with embryogenic competence or not. *CaGH3.15* expression pattern was the only correlated with embryogenic potential and also with the *CaBBM* expression profile, a regulator of somatic embryogenesis in coffee [18, 19] and other species [20]. These findings will help to increase the knowledge about coffee somatic embryogenesis and point to the influence of auxin homeostasis, highlighting molecular aspects that may be useful for the comprehension of this process that could be an alternative for coffee clonal propagation.

## Results

### Identification and distribution of GH3 members in *C. canephora*

The *blastp* analysis against *C. canephora* proteome resulted in 20 amino acid sequences, but three of them (Additional file 1: Data S1) lacked the domains commonly shared by GH3 proteins (PLN02247 and pfam03321). The other 17 putative GH3 members are further summarized (Table 1) with putative protein length, predicted gene position in chromosome (Additional file 3: Figure S1) and locus identification based on Coffee Genome Hub database [21]. Most of these proteins have between 530 and 630 amino acid residues and their genes are distributed along chromosomes 1, 2, 5, 7 and 10. However, almost half of the genes identified in our work are still unmapped (chromosome 0). *CcGH3.10* and *CcGH3.11* are localized in tandem on chromosome 2 and some other genes share high degree of similarity, like *CcGH3.2* and *CcGH3.5* with 98% identity on nucleotide level. In addition, some genes that are not yet anchored to any chromosome seem to be closely mapped in chromosome 0 like *CcGH3.4*, *CcGH3.5* and *CcGH3.6*.

**Table 1:** Description of *GH3* family putative members identified in *C. canephora* through *in silico* analysis

Gene	Conserved domains (CDD - NCBI)	Protein length (aa)	Locus ID (Coffee Genome Hub)	Chromosome position
*CcGH3.1*	PLN02247 superfamily/GH3	593	Cc00_g01360	Chr0: 8,822,291 ... 8,824,450
*CcGH3.2*	PLN02247 superfamily/GH3	583	Cc00_g04490	Chr0: 34,209,474 ... 34,211,882
*CcGH3.3*	PLN02247 superfamily/GH3	371	Cc00_g04500	Chr0: 34,230,766 ... 34,211,882
*CcGH3.4*	PLN02247 superfamily/GH3	583	Cc00_g04520	Chr0: 34,265,456 ... 34,267,828
*CcGH3.5*	PLN02247 superfamily/GH3	583	Cc00_g04530	Chr0: 34,279,931 ... 34,282,340
*CcGH3.6*	PLN02247 superfamily/GH3	357	Cc00_g04540	Chr0: 34,297,176 ... 34,298,627
*CcGH3.7*	PLN02247 superfamily/GH3	569	Cc00_g22520	Chr0: 142,656,917 ... 142,659,058
*CcGH3.8*	PLN02247 superfamily/GH3	348	Cc00_g28980	Chr0: 178,936,581 ... 178,938,006
*CcGH3.9*	PLN02247 superfamily/GH3	606	Cc01_g20620	Chr1: 37,172,864 ... 37,175,503
*CcGH3.10*	PLN02247 superfamily/GH3	271	Cc02_g19460	Chr2: 17,549,243 ... 17,550,429
*CcGH3.11*	PLN02247 superfamily/GH3	236	Cc02_g19470	Chr2: 17,550,591 ... 17,551,298
*CcGH3.12*	PLN02247 superfamily/GH3	399	Cc02_g39050	Chr2: 53,645,460 ... 53,647,471
*CcGH3.13*	PLN02247 superfamily/GH3	607	Cc05_g05640	Chr5: 20,228,391 ... 20,230,769
*CcGH3.14*	PLN02247 superfamily/GH3	591	Cc05_g06700	Chr5: 21,465,217 ... 21,468,524
*CcGH3.15*	PLN02247 superfamily/GH3	622	Cc05_g12940	Chr5: 26,669,847 ... 26,672,091
*CcGH3.16*	PLN02247 superfamily/GH3	528	Cc07_g06610	Chr7: 4,821,858 ... 4,824,041
*CcGH3.17*	PLN02247 superfamily/GH3	583	Cc10_g16320	Chr10: 27,266,812 ... 27,269,856

### Phylogenetic and structural analysis of putative *GH3* genes and proteins

**Fig. 1:**
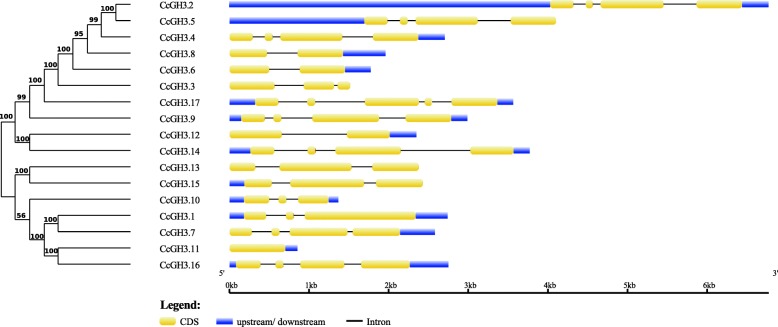
Phylogenetic relationship and genomic structure of *C. canephora* putative *GH3* genes. Exons are represented by yellow ellipses, introns by black lines and upstream/downstream untranslated regions by blue rectangles

All the nucleotide sequences of putative *GH3* genes found in *C. canephora* genome were used as input data to construct a phylogenetic tree (Fig. 1). Some genes have similar genomic structures, although no general structural pattern for *GH3* genes on *C. canephora* was identified.

The tetrad *CcGH3.2*, *CcGH3.4*, *CcGH3.5* and *CcGH3.16* has four exons and three introns with similar lengths. They are similar to the pair *CcGH3.9*-*CcGH3.14*, differing only in intron length. There are other two pairs with similar structure, *CcGH3.6-CcGH3.8* and *CcGH3.13*-*CcGH3.15*. The arrangement of exons and introns did not correlate with similarity at sequence level in all cases, for example, *CcGH3.14* is more similar with *CcGH3.12* at nucleotide sequence level than with *CcGH3.9*.

**Fig. 2:**
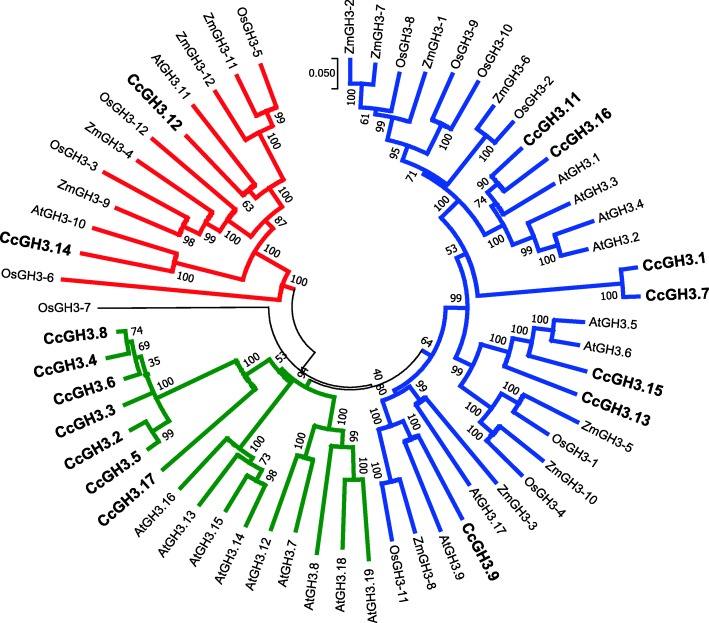
Phylogenetic tree with GH3 proteins from *A. thaliana*, *Z. mays*, *O. sativa* and *C. canephora*. The branches in red, green and blue colors represent the groups I, II and III, respectively

To discriminate *CcGH3s* in functional groups according to literature, a second phylogenetic tree was constructed with GH3 amino acid sequences of * Arabidopsis thaliana*, *Zea mays* and *Oriza sativa* (Fig. 2). This approach clustered proteins CcGH3.12 and CcGH3.14 in group I, CcGH3.2, CcGH3.3, CcGH3.4, CcGH3.5, CcGH3.6, CcGH3.8 and CcGH3.17 in group II and CcGH3.1, CcGH3.7, CcGH3.9, CcGH3.11, CcGH3.13, CcGH3.15 and CcGH3.16 in group III. The OsGH3.7 protein did not cluster with any other sequence, which made it difficult to classify in one of the previous groups. Three sister groups are formed by only CcGH3s and four *C. canephora* GH3 proteins formed sister groups with proteins from other species, which are CcGH3.2-CcGH3.5, CcGH3.1-CcGH3.7, CcGH3.11-CcGH3.16, CcGH3.12-AtGH3.11, CcGH3.14-AtGH3.10 and CcGH3.9-AtGH3.9.

**Fig. 3:**
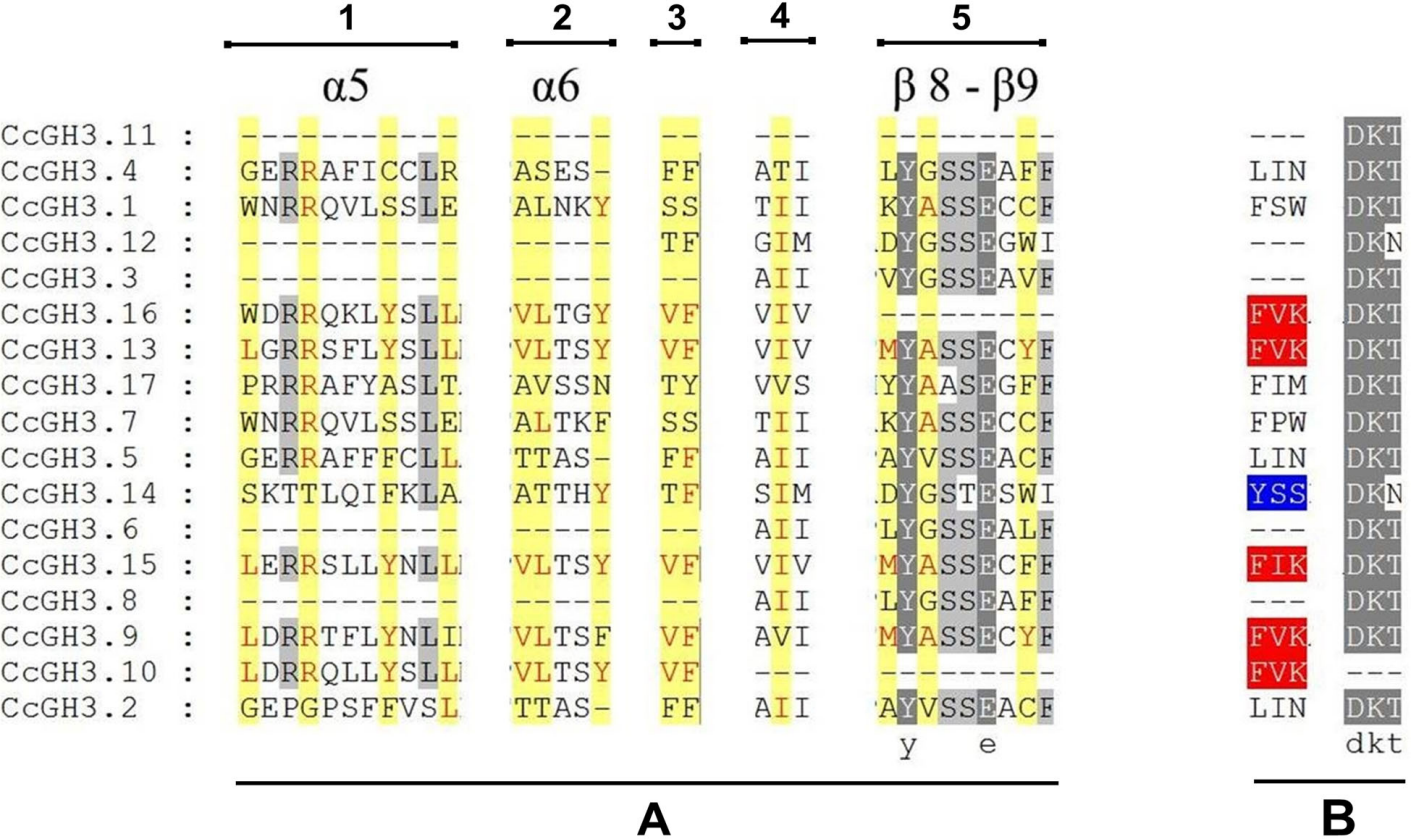
Alignment view of the CcGH3 proteins sections containing the conserved amino acid residues involved in auxin conjugation by acidic amino acids. **a** Alignment of five amino acid residue sets (numbered from 1 to 5) related to acyl acid-binding specificity. The yellow shade represents key residue positions for specificity and residues written in red match with patterns for auxin binding; **b** Set of two amino acid sequences related to amino acid-binding specificity in which red shade represents the pattern for acidic amino acid binding and blue shade represents nonpolar amino acid binding

**Fig. 4:**
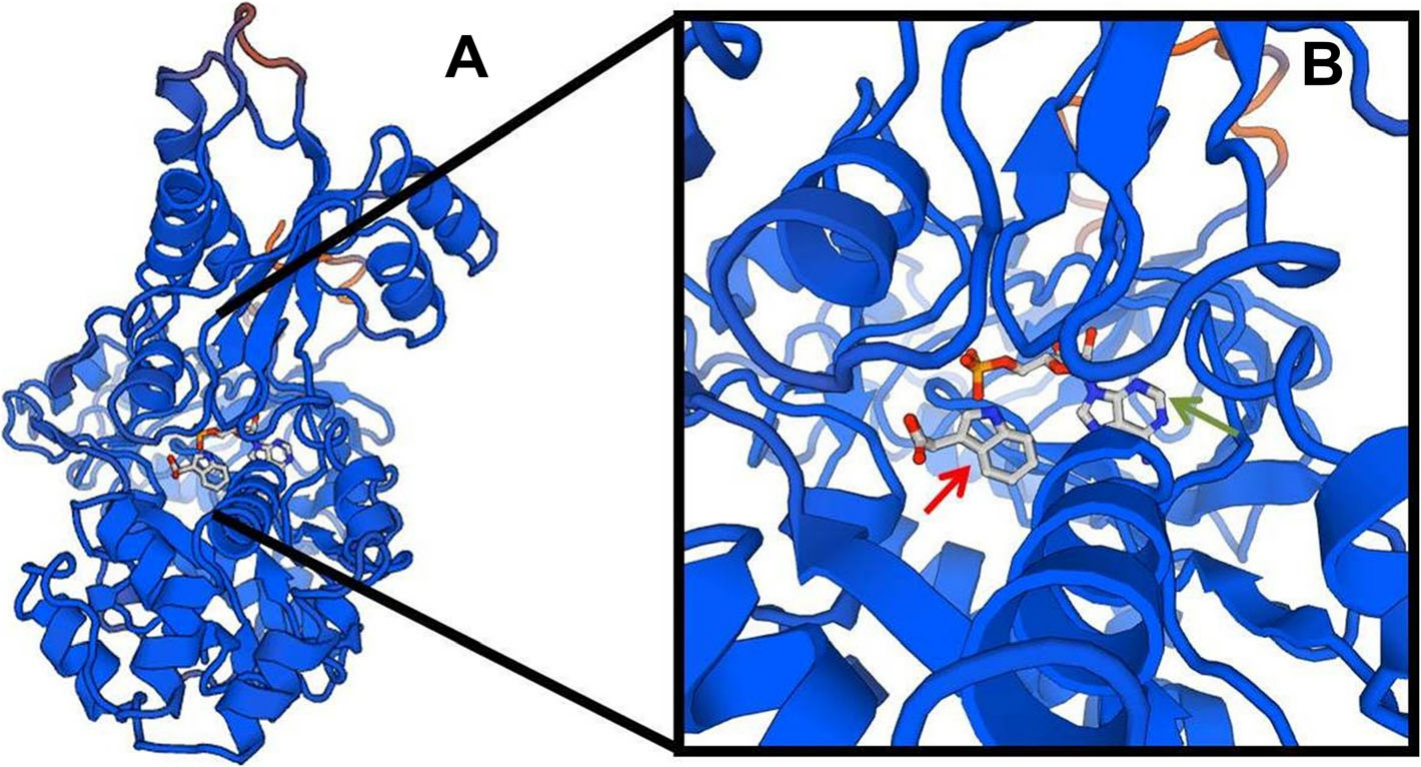
Tridimensional structure of CcGH3.15. **a** overview of the protein structure; **b** close-up to the ligands adenosine monophosphate (AMP, green arrow) and 1H-indol-3-Yacetic acid (IAC, red arrow)

After grouping sequences through phylogenetic relationships, the multiple alignments between all CcGH3 putative proteins were used to search for conserved patterns. Firstly, we searched for sets of amino acid sequences that could be related to acyl substrate specificity as described in literature [4] and afterwards for the sets “F(V/I/T)K” and “DKT”, commonly present in GH3 proteins that conjugate acidic amino acids to auxins [11]. These sequences were found only in CcGH3.9, CcGH3.13, CcGH3.15 and CcGH3.16 (Fig. 3) and such sequences were selected to perform a structural analysis on SWISS-MODEL software [22]. For CcGH3.9, CcGH3.13 and CcGH3.15, the models were constructed based on the crystal structure of GH3.5 of *A. thaliana* [17] and the identities were 55.21, 75.21 and 81.83%, respectively. For CcGH3.16, the best fitted model was based on the crystal structure of a GH3 protein from *Vitis vinifera* [12] with 80.76% identity. In addition to some differences among the four models (Additional file 4: Figure S2), only CcGH3.13 and CcGH3.15 presented ligands like adenosine monophosphate (AMP) and 1H-indol-3-Yacetic acid (IAC) in its tridimensional structure, as further represented for CcGH3.13 (Fig. 4).

### Transcription factors network approach

**Fig. 5:**
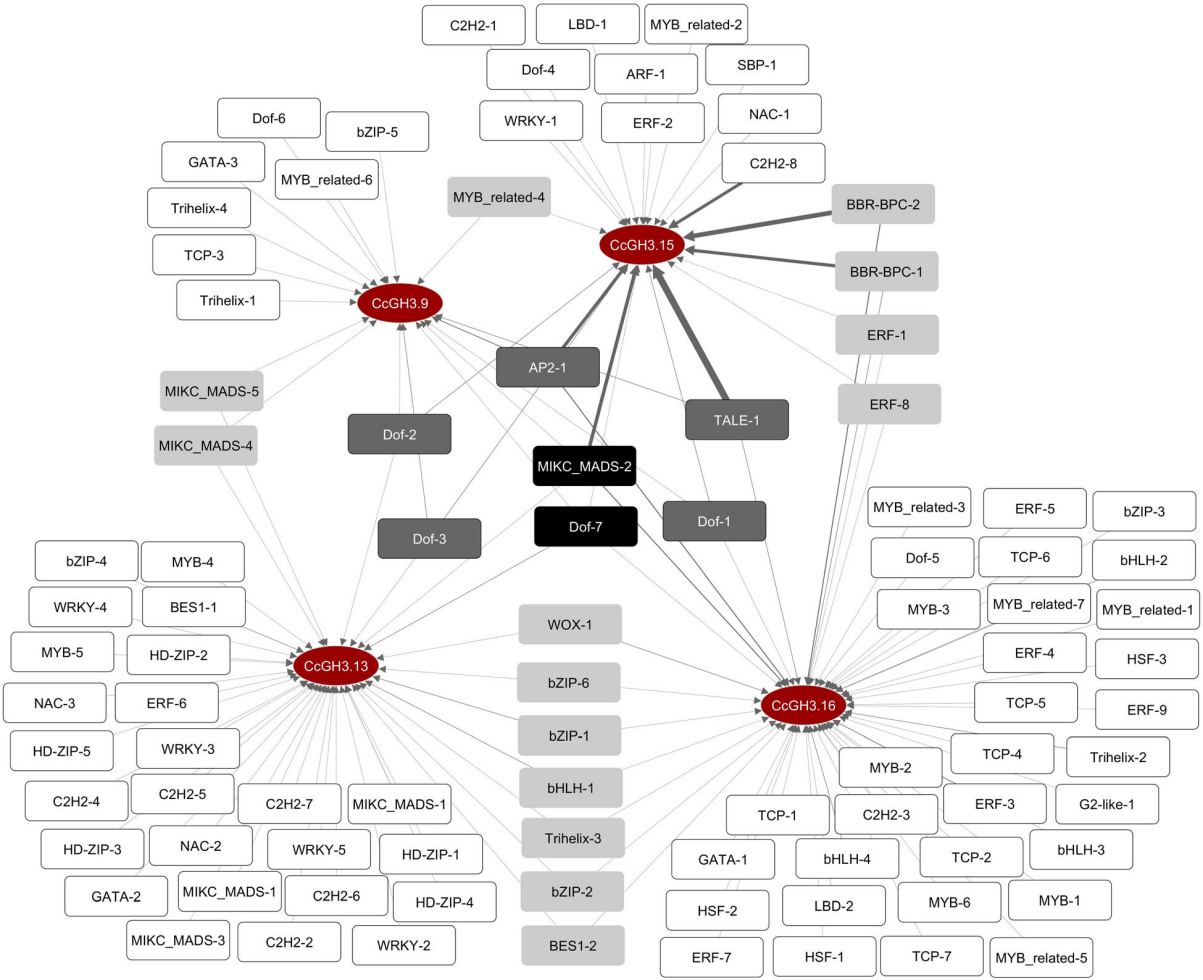
Motif-binding network for selected *CcGH3s* genes (red ellipses) and their related transcription factors (rectangles). The color scale from white to black refers to the number of *CcGH3* genes (one to four) in which a specific transcription factor can bind. Arrow width refers to the number of binding sites for one transcription factor at the promoter region of some *GH3* gene (Additional file 6: Table S1)

An illustrative network between target *GH3* genes and their possible transcriptional regulators was constructed based on data from PlantTFDB [23] about motifs in the *GH3* promoter regions, and named as transcription factors network (Sequences used for constructing the network can be found in the Additional file 2: Data S2). Some motifs are overrepresented in a given promoter and even among *GH3* genes (Fig. 5, Additional file 6: Table S1 and Additional file 7: Table S1 Appendix). The transcriptional regulator with more binding possibilities is the *Cc10_g07850*, a gene from TALE transcription factor family. This gene has 29 binding sites in the *CcGH3.15* promoter and 2 binding sites in both *CcGH3.9* and *CcGH3.16*. Two genes in *C. canephora* genome, *Cc02_g03700* and *Cc07_g05550,* have binding sites in the promoter region of all the tested *GH3* genes and they are members of the MIKC-MADS and Dof transcription factor families, respectively.

The number of motifs found in *CcGH3.9*, *CcGH3.13*, *CcGH3.15* and *CcGH3.16* promoters were 21, 41, 124 and 64, and through these motifs 17, 38, 22 and 48 different transcription factors can bind, respectively. These motifs are specific for genes from 24 different transcription factor families and the ERF family is the most overrepresented. Some families have motifs specific for one out of the *GH3* genes like ARF and SBP for *CcGH3.15*, HSF and G2-like for *CcGH3.16* and HD-ZIP for *CcGH3.13*.

### Histology analysis of *C. arabica* somatic cells with different embryogenic potential and *GH3* gene expression patterns

**Fig. 6:**
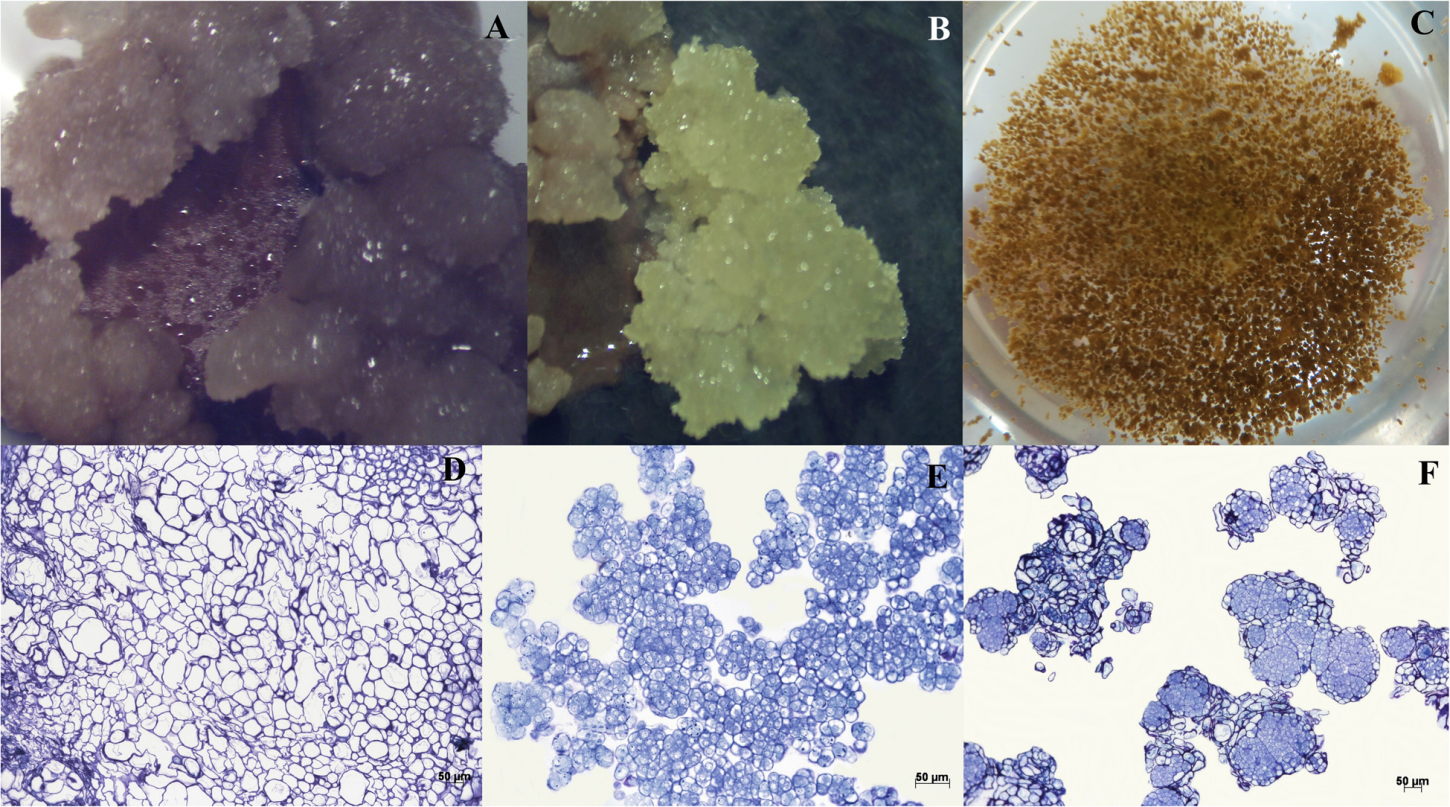
Visual and histological aspects of NEC, EC and ECS. **a** clusters of non-embryogenic cells; **b** clusters of embryogenic cells (the yellow cells are those with embryogenic potential); **c** bottom view of an erlenmeyer containing ECS; Histology of **d** NEC; **e** EC; and **f** ECS, stained with toluidine blue

Embryogenic, non-embryogenic calli and cell suspension with embryogenic potential were sampled for histological and gene expression analysis. The diameter of the different cell types varied between non-embryogenic and embryogenic calli, while the cell suspension with embryogenic potential presented no pattern for cellular length. The cytoplasmic density checked by toluidine blue staining varied regarding the cell types (Fig. 6), in which isodiametric cells were stained more.

For RT-qPCR analysis, the integrity and quality of extracted RNAs were analyzed by electrophoresis and spectrophotometry before its conversion to cDNA. Only samples with suitable characteristics were selected for expression experiments (Additional file 5: Figure S3, Additional file 8: Table S2). All the primers used in RT-qPCR experiments were previously checked for their amplification efficiency with these same samples [18] (Additional file 9: Table S3). A set of candidate reference genes were tested by their stability among the treatment conditions (Additional file 10: Table S4 and Additoal file 11: S4 appendix) and *Ca24S* and *CaRPL39* were established as the most suitable reference genes.

**Fig. 7:**
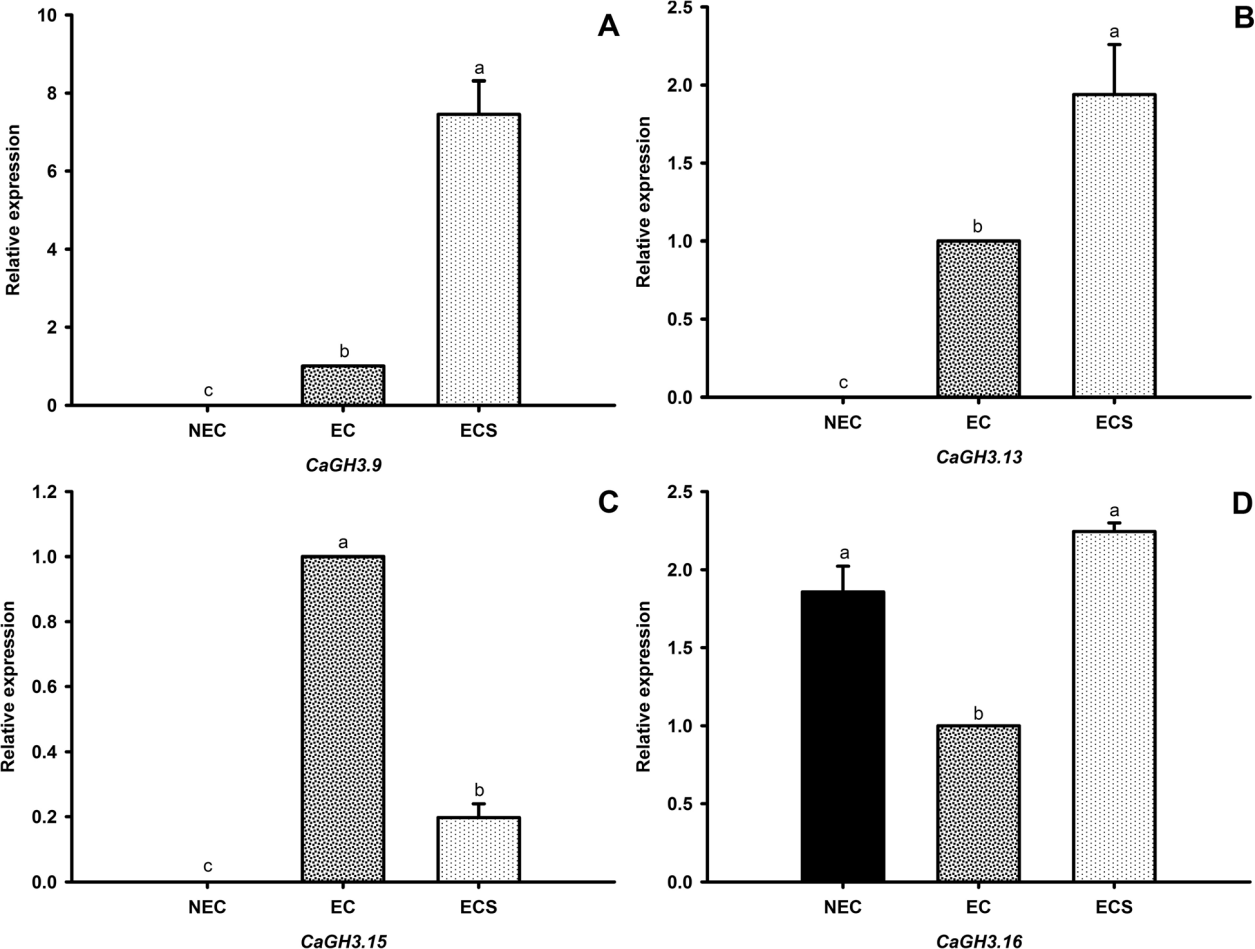
Gene expression patterns of **a** *CaGH3.9*, **b** *CaGH3.13*, **c** *CaGH3.15* and **d** *CaGH3.16* measured by RT-qPCR in NEC, EC and ECS. Data represent mean ± SD. Different letters above columns represent statistical differences among treatments by Tukey test at 5% of significance (*P* < 0.05)

For gene expression analysis, the correspondent genes to *CcGH3.9*, *CcGH3.13*, *CcGH3.15* and *CcGH3.16* in *C. arabica* (*CaGH3.9*, *CaGH3.13*, *CaGH3.15* and *CaGH3.16*, respectively) were selected (Fig. 7), based on previous results demonstrating their possible involvement in acidic amino acid conjugation to auxin. *CaGH3.9*, *CaGH3.13* and *CaGH3.15* did not present any expression in non-embryogenic cells (NEC). *CaGH3.9*, *CaGH3.13* and *CaGH3.16* presented higher expression in embryogenic cell suspension (ECS), while *CaGH3.15* had more transcript quantity in embryogenic cells (EC). The *CaGH3.16* gene was the only that exhibited expression in all the cell types.

## Discussion

The *C. canephora GH3* putative genes identified in our work have all the conserved domains commonly found in members of this family. Such domains are required for its proper functionality and the number of putative *GH3* members found herein is close to other dicotyledonous species like *Malus domestica* [15], *Medicago truncatula* [16] and *Solanum lycopersicum* [14]. However, *C. canephora* did not go through any polyploydization event after core eudicots diversification, unlike *S. lycopersicum*, which belongs to the same *C. canephora* class (asterid) [21]. Therefore, it seems some *CcGH3s* could have been originated from local duplications.

This hypothesis is interesting upon analysis of *C. canephora GH3* genes containing similar structures, like the tetrad *CcGH3.2*, *CcGH3.4*, *CcGH3.5* and *CcGH3.16* and the pair *CcGH3.8* and *CcGH3.6* (Fig. 1). Except for *CcGH3.16*, the other genes are clustered in a group exclusively constituted by *C. canephora* GH3 proteins in the phylogenetic tree, constructed with GH3 protein sequences of *A. thaliana, O. sativa* and *Z. mays* (Fig. 2). These proteins from *C. canephora* are the closest in sequence similarity to those from *A. thaliana* apparently local duplicated in this species [13]. Further detailed syntenic studies may confirm such hypothesis and help to understand if there is a specific function evolved in *C. canephora* for these members of GH3 family.

Studies supported on the gene family wide analysis approach have been broadly performed recently [24–27]. These studies have been also applied to unravel *GH3* gene family members characteristics in a wide perspective, usually with genic and protein structure description, gene expression patterns along plant tissues [28] or analyzed in a specific process [29]. Here, we speculate if some genes of *GH3* family may influence the somatic embryogenesis in coffee tree, specifically the possible correlation with embryogenic potential of different types of calli, which is the key to understand indirect somatic embryogenesis process.

Group III from the phylogenetic tree has the most widely studied members and all the *A. thaliana* proteins clustered are associated to amino acid conjugation to auxin, accordingly to transcriptional activation, enzyme activity or mutant phenotype assays [13]. The involvement of some CcGH3 homologs in the conjugation of auxins to amino acids can be analyzed through reports in the literature such as for the members AtGH3.2, AtGH3.3, AtGH3.4, AtGH3.5 and AtGH3.6 [30] and AtGH3.9 [31]. These works have suggested that in the presence of GH3 family members auxins can be conjugated to different amino acids using specific approaches to analyze the conjugation product. Furthermore, studies on the 3D molecular structure of AtGH3.5 followed by *in vitro* and *in planta* biochemical analyses suggest the ability of this protein in conjugating auxins to amino acids and mediate their homeostasis [17]. This reinforces the importance of investigating some CcGH3 members clustered together with these well-studied AtGH3 proteins and to link the role of conjugating auxin with the coffee somatic embryogenesis.

Amino acid sequence alignment with functional characterized proteins revealed a correlation between substrate specificity and conserved sequence patterns [11, 17]. It allowed us to choose four CcGH3s candidates likely involved in acidic amino acid conjugation to auxin, such as the members CcGH3.9, CcGH3.13, CcGH3.15 and CcGH3.16. Although just CcGH3.13 has all conserved residues for both auxin and acidic amino acid binding sites, the CcGH3.9 and CcCGH3.15 proteins have sufficient potential to be further analyzed as well, as the present mismatches do not change the amino acid classes and, also, we decided analyze CcGH3.16, besides the absence of amino acids residues in positions of β8-β9 (Fig. 3).

The tridimensional models for the four CcGH3s proteins revealed that only CcGH3.13 and CcGH3.15 have AMP and IAC ligands (Fig. 4, Additional file 4: Figure S2). The binding of IAC ligand represents the affinity for auxin as a substrate for this enzyme and the interaction is mediated by the highlighted amino acid residues sites of α5, α6 and β8-β9 (Fig. 3a) [17]. In fact, the IAC ligand interaction in tridimensional model is supported by the presence of the correct set of amino acid residues in the interaction sites, by comparison with GH3 proteins that are already confirmed as having auxin affinity [17]. This suggests the potential of these proteins to influence auxin homeostasis dependent process in the plant.

To further speculate on whether the selected *GH3* members have a possible link to cell embryogenic competence, we identified the binding sites of transcription factors in promoter regions of these four *CcGH3s* genes and with this, a transcription factor network was constructed aiming to analyze the transcription factor binding possibilities in each gene. The network was constructed based on information taken from Coffee Genome Hub and PlantTFDB databases [21, 23] to support the transcription factor possible connections for each selected *CcGH3* gene. Genes from 24 different transcription factor families were identified as able to bind in the *GH3* promoters. Out of 24, at least 15 families have members with already known influence on somatic embryogenesis process [9, 32, 33].

The Ethylene Responsive Factor (ERF) family, the most overrepresented transcription factor family in the network with 9 genes, has members already reported to influence on somatic embryogenesis [9], like *SERF* in *Medicago truncatula* and *ERF022* in *A. thaliana.* Furthermore, a decrease in ethylene concentration was observed in calli from a maize lineage with high embryogenic potential, likely regulated by an ERF gene, which did not occur with the low embryogenic potential lineage [34].

Another interesting feature in the network is that some genes contain multiple binding sites at certain promoters, what could influence still more upon its probability of regulating specific gene expression, like TALE-1 (*Cc10_g07850*), that have 29 predicted binding sites in *CcGH3.15* and just two in *CcGH3.9* and *CcGH3.16* promoters. This transcription factor belongs to TALE family, subfamily KNOX, which is reported to have members involved in the shoot apical meristem maintenance [35, 36]. Although we cannot speculate at this moment if this transcription factor is an activator or repressor of *CcGH3.15*, a regulatory influence is reinforced by the number of binding sites in the *CcGH3.15* promoter, which may be associated to meristem-like features of embryogenic cells. Furthermore, in japonica rice subspecies, the downregulation of a KNOX-type transcription factor improved the regeneration and development of somatic embryos, highlighting the additional role of such subfamily on the somatic embryogenesis process [37].

Taking into account the differences in regulation profile of each *CcGH3* proposed by the network, we performed an RT-qPCR experiment to analyze the expression pattern of these genes in *C. arabica* cell types contrasting in its embryogenic potential. The cell types used are clearly different; those with embryogenic potential are isodiametric, have larger nucleus and high cytoplasmic density, which are the expected characteristics for embryogenic coffee cells [19, 38, 39]; whereas the non-embryogenic ones are larger, with undefined shape and low cytoplasmic density [40]. The embryogenic cell suspension is composed by these two cell types, an interesting material for correlating embryogenic potential to gene expression pattern.

It is reported that AtGH3.17 protein inhibits hypocotyl cell elongation by means of auxin conjugation with glutamate [10], indirectly influencing over hypocotyl cell expansion through H^+^- ATPase proton efflux [41]. Accordingly, we speculate that the embryogenic and non-embryogenic cell shapes presented in histological analysis could be a consequence of GH3-mediated auxin conjugation, what would reinforce the influence of this protein over coffee early somatic embryogenesis process.

Although we selected just *GH3* genes similar in amino acid sequence level, they exhibited a different expression pattern among the samples, possibly due to different roles performed by these enzymes in metabolic pathways and their requirements within the cellular environment. Even being descendent from a common ancestral, paralog genes tend to perform distinctive functions in a given organism. It is consistent with the patterns of gene family evolution, which is generally owing to duplications (either local or derived from whole genome duplication), neo/subfunctionalization and retention according or not to environment selective pressure [42].

*CaGH3.9* and *CaGH3.13* had similar expression patterns among cell types (ρ = 0,9393, correlation by Spearman method, Additional file 12: Table S5), but *CaGH3.13* does not share many transcription factor binding sites with *CaGH3.9* like it shares with *CaGH3.16* (Fig. 5). The predicted transcription factor binding sites are condition-independent, thus not all of them necessarily regulate the *GH3* genes at certain tissue or timing. We speculate that *CaGH3.9* and *CaGH3.13* expression pattern similarities in our embryogenic cell types may be owing to shared binding sites, even if a greater number of unshared motifs can influence transcriptional regulation in other conditions. The common binding sites of these two genes are for the transcription factors *Cc11_g17110* and *Cc10_g13750*, both belonging to MIKC-MADS family, which has already characterized members related to many regulatory functions in plant kingdom [43], including some that influence over somatic embryogenesis [44].

*CaGH3.15* gene has the most correlated expression pattern with embryogenic potential of cell types tested: high transcript levels in embryogenic cell, average values in embryogenic cell suspension (which is composed by both embryogenic and non-embryogenic cells) and lower expression in non-embryogenic cells. This gene also exhibited the most evident relationships with some regulators in the network, due to high number of binding sites for a specific transcription factor. While other *GH3* genes have in maximum four binding sites for a given transcription factor, *CcGH3.15* have 29, 20, 15, 14, 14 and 13 binding sites for the transcription factors TALE-1, BBR-BPC-2, MIKC-MADS-2, BBR-BPC-1, AP2–1 and C2H2–8, respectively.

The protein families TALE, MIKC-MADS, AP2 and C2H2 have well characterized TFs involved in somatic embryogenesis while BBR-BPC, despite being a poorly explored TF family in plants, has a member reported to control floral meristem size and seed development by regulating other transcription factors like LEC2 and WUSCHEL [20, 45]. These last two genes have been considered key regulators of somatic embryogenesis [9], thus BBR-BPC genes might also be involved in SE process.

The *Cc09_g04020* gene belongs to AP2 family and is the most suitable transcription factor that can explain the *CaGH3.15* expression pattern in cells with embryogenic potential. This *C. canephora* gene is the most similar to the previously characterized *BBM* gene from *C. arabica,* which was already studied during embryogenic process [18, 19]. Recently, this gene was reported to be the major regulator of somatic embryogenesis, being associated to cellular totipotency induction through the control of *LAFL* (*LEC1*, *ABI3*, *FUS3*, LEC2) gene network [46].

**Fig. 8:**
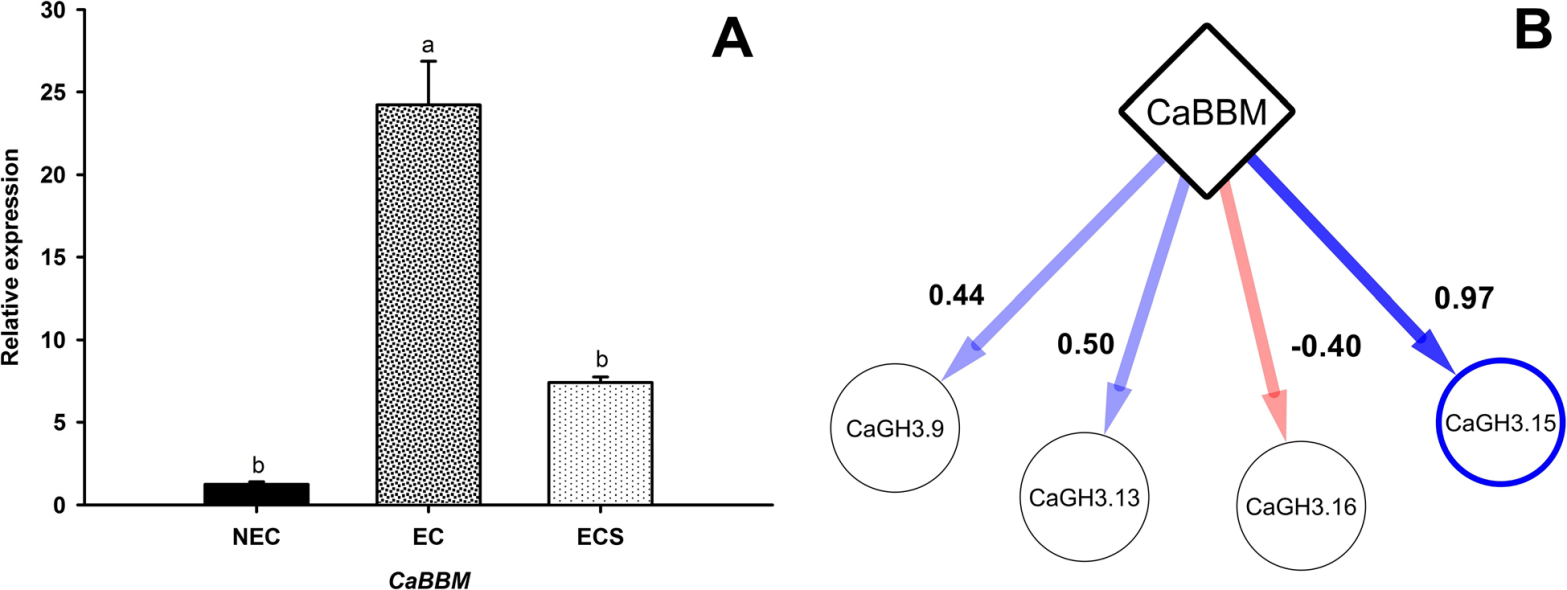
Expression profile of *CaBBM* and its regulatory relationships with *CaGH3.9*, *CaGH3.13*, *CaGH3.15* and *CaGH3.16.* **a** Expression pattern of *CaBBM* in non-embryogenic cells (NEC), embryogenic cells (EC) and embryogenic cell suspension (ECS) measured by RT-qPCR; **b** Co-expression analysis of *CaBBM* gene with *CaGH3.9*, *CaGH3.13*, *CaGH3.15* and *CaGH3.16,* inferred by Spearman’s method; dark blue means significant positive Spearman’s correlation, light blue non-significant and light red non-significant negative Spearman’s correlation

We reinforced this regulation hypothesis for the *CcGH3.15* by comparing through the Spearman’s correlation method the gene expression data of *CaGH3s* members with previously published data from our group for the *CaBBM* [18] gene. A strong correlation value was found (ρ = 0.971) only for *CaGH3.15* while the maximum correlation value observed for the other *GH3* genes was ρ = 0,50, meaning that only *CaGH3.15* and *CaBBM* are co-expressed in these conditions (Fig. 8).

We suggest that the *CaGH3.15*, one of the members of *GH3* gene family in *C. arabica*, could be regulated by *CaBBM*, and it possibly influences auxin homeostasis during somatic embryogenesis in coffee. This is consistent with the higher IAA-Glu conjugate concentration in comparison to free auxin and IAA-Ala detected during somatic embryogenesis induction in *C. canephora*, which reflects the requirement of decreasing auxin content during somatic embryogenesis process [47].

In *C. canephora*, the somatic embryogenesis happens directly in the explant, without an embryogenic calli formation step. The auxin content decreases during the induction phase, what may also be due to its transport to explant extremities, where the embryos need this phytohormone supply to develop [48]. The process in *C. arabica* is through indirect embryogenesis, with the need of embryogenic calli induction before somatic embryo regeneration, and two types of auxins were included in the medium [40]. Embryogenic and non-embryogenic calli derive from the same explant (leaf cuts, Fig. 6b), but those without embryogenic competence appear earlier, sometimes with more than two months of difference. Therefore, auxin homeostasis through amino acid conjugation, which we suggest that can be an effect of *CaGH3.15* upregulation demonstrated in this work, might be a differentiation factor between these two types of calli and necessary for embryogenic competence achievement of the coffee cells.

Somatic embryogenesis is a bottleneck process for *C. arabica* clonal propagation and genetic transformation. Although many breakthroughs were achieved recently to scale this technology to commercial level, the improvements are mostly due to empirical research and key points of process yet remain unclear, with the need of wider cellular and molecular comprehension [49]. Here, we identified a *GH3* gene member potentially associated to early somatic embryogenesis process in *C. arabica*, which may have influence on auxin degradation, due to conjugation with acidic amino acids. Further analysis needs to be performed to unravel the many aspects related to SE at cellular and molecular level, to deepen the understanding of this process and establishing an efficient clonal propagation protocol based on totipotent cell regeneration, which is essential in the pursuit of this crop ideotypes in genome editing era [50].

## Conclusions

Here we report that the GH3 family is conserved in *C. canephora*, with some of the members clustered by similarity at amino acid sequence level with proteins already known to be associated to auxin conjugation. Four of these coffee GH3 members have most of the conserved amino acid sets related to acidic amino acid conjugation to auxin. Besides structural properties, one out of all *CcGH3* members, the *CcGH3.15*, have binding sites in its promoter region for some transcription factors that might be associated to embryogenic features.

The homolog of *CcGH3.*15 in *C. arabica*, named *CaGH3.15*, has the transcriptional profile most correlated with embryogenic competence, addressed here by using coffee cells with contrasting embryogenic potential, and correlation with the expression profile of *CaBBM*, the closest coffee homolog to *BBM* transcription factor, a major plant embryogenesis regulator. All these findings together indicates that *GH3* genes may have influence on the internal fine tuning of auxin content in the cell, necessary for totipotency acquirement and maintenance in the embryogenic competent cells. As the coffee market mostly relies on this species cultivation, all knowledge and the available tools need to be explored to ensure a sustainable coffee production chain.

## Methods

### Identification, phylogenetic and structural analyses of GH3 members in *C. canephora*

The GH3 amino acid sequences of *A. thaliana*, *Z. mays* and *O. sativa* were used as query for blastp analysis against *C. canephora* proteome in Coffee Genome Hub Database^19^, with e-value threshold of 1.0 × 10^− 5^. The resulting sequences were then submitted to NCBI Conserved Domains Database (NCBI-CDD) aiming to verify the presence of conserved domains presented in proteins of this family, PLN02247 and pfam03321. The sequences that satisfied this requisite were considered as putative GH3 members.

The genomic sequences of these putative GH3 members were submitted to MEGA 7.0 software [51] to perform an alignment by ClustalW algorithm [52] and phylogenetic analyses by Neighbor-joining [53] and p-distance [54] methods, with 1000 bootstrap replications. The resulting file with phylogenetic relationships, together with coding and genomic sequences of *GH3* genes were used to design a graphical representation of gene structure with phylogenetic relationships in Gene Structure Display Server (GSDS 2.0, http://gsds.cbi.pku.edu.cn/) [55]. A second phylogenetic tree, constructed with same software and by same methods, was based on amino acid sequences of putative *C. canephora* GH3s and those from *A. thaliana*, *Z. mays* and *O. sativa*, aiming to speculate CcGH3s phylogenetic relationship. The proteins AtGH3.20 and CcGH3.10 were excluded from the phylogenetic tree because the pairwise distances of some pairs that included these proteins were not calculated by the program (MEGA7) with the parameter we have set.

The putative *C. canephora* proteins were also aligned for analysis of conserved sets of amino acids related to substrate specificity [11]. Finally, the four selected putative CcGH3 proteins were submitted to Swiss-model software (http://swissmodel.expasy.org/) [56], in which CcGH3.9, CcGH3.13 and CcGH3.15 tridimensional structures were modeled based on GH3.5 of *A. thaliana* (5kod.2.A template) and CcGH3.16 based on GH3 protein from *Vitis vinifera*, all built with ProMod3 version 1.1.0.

### Motif-bind network construction

The upstream regions (3 kb before ATG) from *C. canephora GH3* genes *CcGH3.9*, *CcGH3.13*, *CcGH3.15* and *CcGH3.16* were downloaded from Coffee Genome Hub database (CGH) and were submitted to regulation prediction on Plant Transcription Factor Database 4.0 (http://planttfdb.cbi.pku.edu.cn/) [23] to predict which transcription factors can bind to each GH3 promoter. The detected genes were then used as source nodes, the *GH3* genes as target nodes and the number of motifs found in each GH3 promoter for each gene was used as a score to construct a network. With the locus id of each detected gene, their amino-acid sequences were downloaded from CGH, submitted to TF prediction server and this classification in transcription factor family was used for the network enrichment. Finally, the transcription factors were also discriminated by the number of possible interactions with *CcGH3s* to enrich the network.

### Achievement and histology of embryogenic, non-embryogenic and suspension cells

Leaves of greenhouse grown *C. arabica* cv. Catuaí Amarelo IAC 62 plants were used as explants for embryogenic, non-embryogenic calli and embryogenic cell suspension achievement, accordingly to an already established protocol [57]. The embryogenic cell suspension was cultivated in Erlenmeyer flasks with T3 medium [58], with initial density of 10 g L^− 1^ in absence of light at 25 °C and 100 rpm.

Samples of the plant materials were fixed with FAA_70_ (10% formaldehyde + 5% acetic acid + 70% ethanol, v/v) for 48 h at room temperature, dehydrated in a graded series of 60, 70, 80, 90 and 100% ethanol. Then the samples were embedded in epoxy resin (*Historesin*® *Leica*) according to manufacturer’s protocol. The blocks were sectioned into 2 μm slices using manual rotary microtome (*Easypath* EP-31-20,091), stained with 0.05% toluidine blue and analyzed with a light microscope (*Zeiss*, *Axio Scope*). Images were analyzed using *AxioVision* 4.8 capture system.

### RT-qPCR analysis of putative *GH3* genes and correlation with *CaBBM*

Total RNA was extracted from non-embryogenic calli, embryogenic calli and embryogenic cell suspension (3 months) using Invitrogen (Life Technologies, Carlsbad, CA, USA) Concert™ Plant RNA reagent. RNA extracts were treated with Ambion (Life Technologies) Turbo DNA-free kit reagents in order to remove any genomic DNA. The quantity and purity of total RNA was assessed with an ND-1000 spectrophotometer (NanoDrop Technologies, Wilmington, NC, USA). The synthesis of cDNA from 1,000 ng aliquots of RNA was carried out using Applied Biosystems (Life Technologies) High-Capacity cDNA Reverse Transcription kit according to the recommendations of the company.

RT-qPCR analyses were performed using an Applied Biosystems 7500 Real-Time PCR system with a reaction mix containing 5.0 μL of SYBR® Green PCR Master Mix (Applied Biosystems), 2 ng of cDNA, 0.4 μM of primers and RNase-free water to a total volume of 10 μL. Amplification conditions were initial activation at 95 °C for 10 min and 40 cycles of denaturation at 95 °C for 15 s and combined annealing and extension at 60 °C for 1 min. The primers design were suitable to RT-qPCR technique and the amplification of both homeologs in *C. arabica* genome, derived from *C. canephora* and *C. eugenioides*, was assured through analysis of the sequences relative to *CcGH3* genes in Phytozome database. (https://phytozome.jgi.doe.gov/pz/portal.html#!info?alias=Org_Carabica_er) using blastn tool and analysis of multiple alignments performed by ClustalW [52] algorithm and visualized by GeneDoc software [59]. Expression data were normalized with reference genes *24S* and *RPL39* and relatively quantified by applying Pfaffl method [60]. Shapiro-Wilk test was performed to verify the normality of data distribution. Data that did not fit into test’s prerequisites were transformed with the method [log10(x + 0.5)]. Statistical data analysis was performed by two-way ANOVA upon three biological replicates (*n* = 3) per sample followed by a comparison among gene expressions for each *GH3* gene member at the cell types using the Tukey’s test at 5% significance (*P* < 0.05).

The co-expression analysis was made by using the expression profile of *CaGH3.9, CaGH3.13*, *CaGH3.15*, *CaGH3.16* genes together with *CaBBM* [18], with all the data obtained in the same samples, with the same reference genes and equipment. The correlation values were generated by Spearman’s correlation analysis using R software.

## Supplementary information

**Additional file 1:** **Data S1.** Amino acids sequences related to GH3 proteins in *C. canephora* available at Coffee Genome Hub website. Three (Cc00_g28970, Cc02_g39040 and Cc00_g04550) out of the twenty amino acid sequences lacked the domains commonly shared by GH3 proteins (PLN02247 and pfam03321); therefore these sequences were not selected for analysis.

**Additional file 2:** **Data S2.** Complete set of amino acid sequences used into the network analysis.

**Additional file 3: Figure S1.** Gene distribution of *CcGH3* members on *C. canephora* chromosomes using the option “locus search” in the Coffe Genome Hub website. The colors within each chromosome indicate ancestral blocks corresponding to the 7 core eudicot chromosomes (DENOUED et al., 2014). Red lines stand for the position of each *CcGH3* over chromosomes. The most *CcGH3* members are not presented in any chromosome, so they were allocated in chromosome 0 (not available). The genes are distributed as follow into each chromosome (chr): chr1: *CcGH3.9*; chr2: *CcGH3.10*, *CcGH3.11* and *CcGH3.12*; chr5: *CcGH3.13*, *CcGH3.14* and *CcGH3.15*; chr7: *CcGH3.16*; chr10: *CcGH3.17*.

**Additional file 4:** **Figure S2** Tridimensional structure model for the four GH3 proteins selected in *Coffea canephora*.

**Additional file 5:** **Figure S3** RNA integrity assessment by agarose gel electrophoresis. This figure was cropped from the original picture to exhibit the RNAs extracted from three biological replicates referred to each cell type used for RT-qPCR analysis. The bands clearly separated and visible represent two rRNA subunits point to the good RNA quality. From the left to the right are the samples: Embryogenic cell suspension, biological replicates 1, 2 and 3; Embryogenic calli, biological replicates 1, 2 and 3; Non-embryogenic calli, biological replicates 1, 2 and 3.

**Additional file 6:** **Table S1** Number of binding possibilities for each transcription factor in selected GH3 promoter regions.

**Additional file 7:** **Table S1 Appendix.** Description of binding possibilities for each transcription factor in selected *GH3* genes promoter regions.

**Additional file 8:** **Table S2** Concentration and quality assessment of RNA samples used for RT-qPCR analysis.

**Additional file 9:** **Table S3** Primers used in RT-qPCR experiments.

**Additional file 10:**: **Table S4** Endogenous gene expression stability analyzed by RefFinder software (http://fulxie.0fees.us/).

**Additional file 11:** **Table S4 Appendix.** Primer pairs used into analysis of expression stability for endogenous genes (Freitas et al. 2017).

**Additional file 12:** **Table S5** Gene expression profile correlations between *CaGH3s* and *CaBBM*. Scores were obtained by Spearman’s correlation.

## Data Availability

All amino acid sequences used into the network analysis are available on the Supplementary data 2. Embryogenic and non-embryogenic calli herein used were obtained under in vitro culture conditions previously described in the materials and methods. The primer sequences for amplification of the *GH3* family genes are also available at the Supplementary files. In case of any information required for reproduction of the experimental data is not present elsewhere within publication content, please directly contact the corresponding authors on reasonable request.

## Abbreviations

AMP: Adenosine monophosphate
ANOVA: Analysis of Variance
AP2: Transcription factor with Apetala2 domain
ARF: Auxin response factor
BBM: Baby Boom
BBR-BPC: BBR-Basic pentacysteine
Blastp: Basic Local Alignment Search Tool (proteins)
C2H2: Trancription factor encoding C2H2 zinc finger domains
CDD: Conserved domain database
cDNA: Complement DNA
CGH: Coffee Genome Hub
Dof: DNA binding with one finger
EC: Embryogenic cells
ECS: Embryogenic cell suspension
ERF: Ethylene Responsive Factor
FAA: Formaldehyde,Acetic acid, Alcohol (ethanol)
G2-like: Golden 2-like
GH3: Gretchen Hagen 3
GSDS: Gene Structure Display Server
HD-ZIP: Homeodomain and leucine zipper containing transcription factors
HSF: Heat stress transcription factors
IAA: Indole-3-acetic acid
IAC: 1H-indol-3-Yacetic acid
KNOX: Knotted-like domain containing transcription factors
LAFL: (LEC1, ABI3, FUS3, LEC2)
LEC2: LEAFY COTYLEDON 2
MIKC-MADS: MIKC-type MADS box
NCBI: National Center for Biotechnology Information
NEC: Non-embryogenic cells
PlantTFDB: Plant Transcription Factor Database
RT-qPCR: Quantitative reverse transcription Polymerase Chain Reaction
SBP: Squamosa promoter binding proteins
SD: Standard deviation
SE: Somatic embryogenesis
TALE: Transcription factor containing from theThree-amino-acid-loop-extension class
TF: Transcription factor
