# Supplementary material for: Genome-wide analysis, transcription factor network approach and gene expression profile of GH3 genes over early somatic embryogenesis in Coffea spp
Source: BMC Genomics. 2019 Nov 6;20:812. doi: 10.1186/s12864-019-6176-1 (PMC6836404; doi:10.1186/s12864-019-6176-1)
Supplement: Supplementary file 1 — Additional file 1: Data S1. Amino acids sequences related to GH3 proteins in C. canephora available at Coffee Genome Hub website. Three (Cc00_g28970, Cc02_g39040 and Cc00_g04550) out of the twenty amino acid sequences lacked the domains commonly shared by GH3 proteins (PLN02247 and pfam03321); therefore these sequences were not selected for analysis. [file 12864_2019_6176_MOESM1_ESM.pdf]

Supplementary data 1. Amino acids sequences related to GH3 proteins in *C. canephora* available at Coffee Genome Hub website. Three (Cc00_g28970, Cc02_g39040 and Cc00_g04550) out of the twenty amino acid sequences lacked the domains commonly shared by GH3 proteins (PLN02247 and pfam03321); therefore these sequences were not selected for analysis.

>CcGH3.1
METDWNTKALEFIEEMTKNAGEVQKMVLADILKQNGETEYLQRFNLDGATDTKTFTSKVPCITY
EDIRPEIQRMENGDRSAILTALPVSDFLLSSGTTSGKRKLIPMPEEEWNRRQVLSSLEIPVMNVYV
PDLNRGKGLYFYFSWPEIRTPGGQMILTALNKYYRSEHFKNQRRDPYTQYTSPYESVLCTDYVQ
SMYVQLLCGLYQHKQINRVGASSASALLRVIKFFELNWQDLVLDIRVGSLNPKITYEPLRECMAR
IMKSDPELADFLTIECSGENWEGIIQRIWPNAKYLGTIITGSMIQYAPSLDYYSGGLPIATSKYASS
ECCFGINLNPISKPEDVSFTFMPNLAYFEFIPQEHHSSNMDIGNSTVTTPDLINLVDVEVGKDYEV
VITTHAGLYRYQMGDILRVTGFHNSAPKFKFLRRKGVLLSIAGDKTDEVELQMAMDNASQILQE
YSVSLVDYTSHASKKIAPGHYVIYWELSVGVLGDSDKNAQSDEVISRCCPIIENSFSLIYKQHRVH
GAIAALEIRVLKNGTFQDLVEFAVSRGASIGQYKVPRCVETGPVLEFLDSRVVSSHFSPCLPSLAS
EESI*
>CcGH3.2
MLPTFDPRDTEAGSRILEDITSNAGHIQEQVLEEILTKNASTDYLKSFLNGHSDKGLFKNKVPVVD
YEDIKIYIDRIALDGEPSRILTNESITELMISSGTSGGKQKWIPKTAEEGEPGPSFFVSLLLFELRCLH
GLNDGKALFFVLINPDIHTPGGLVLRTTTASEIKNRKDRYPEVILCEDTNQSLYSQLLCGLVQRDA
IVSIGTFFASGLQRIIKFFEEHWQEMSSNIRTGQMSDWITDPKCKRTVSLILSKQMPDLADSIDLVC
QEKSWEGIIKKIWPRTKYVQAIITGSMAQYIPALEFYTGRLPVVSPAYVSSEACFGVNFKPLCNPY
DVSYTFIPNMAYYEFIPIGNHQDPNCTNSKDAHLKDHIVDLANVKIGQHYELLVTTCTGLYRYR
MGDILLVTGFHNITPQFKFVQRRNVVLSIDTDKTTEQDLQNAVTIAMHILEPLGFFLLDYSSYADT
SSIPGHYVLFWELQLRSNNDIPELDQVKMEKCCSLVEQSLDQKYKLLRNQSISTIGPLEIRVVKQG
TFNVLMDFYVSRGTSLNQYKTPKNIKSEKAIEILDSRVVGKFYSREVPNQDS*
>CcGH3.3
MTYGGFQGASYDIWVIKFFKENWQEMSSNIRTGQMSDWITDPNCKRAVSLILSKQMPDLADSID
RVCQEKSWEGIIKKIWPRTKYVMAIITGSMAQYIPALEVYTGGLPVVSPVYGSSEAVFGINMKPL
CRPYDVSYTFIPNMAYYEFLPIDNHQDPNCTIRKDAHLKDHIVDLANVKIGQHYELLVTTRVLSG
LYRYRMGDILLVTGFHNITPQFKFVQRRNVVLSIDTDKTTEQDLQNAVTIAMHILEPLGLFLLDYS
SYADTSSIPGHYVLFWELQLRSNDDFPVLDQVNMEKCCSLVEQSLDQKYKLLRNQSIRTFNVLM
DFYVSQGTSLNQYKTPKNIKSEKAIEILDSRVVGKFYSREVPNQDS*
>CcGH3.4
MLPTFDPTDTEAGSRILEDITSNAGHIQEQVLEEILTKNASTDYLKGFLNGHSDKGLFKNKVPVV
DYEDIKIYIDRIALDGEPSRILTNESITELLKSSGTSGGKQKWIPKTAEEGERRAFICCLRDTVLNRY
LQGLSDGKALLFVLINPDIHTPGGLVLRTASESEIKNRKDRYPQFILCEDTNQSLYGQLLCGLVQR
DAIVSVGTFFASGLLRIIKFFEEHWQEMSSNIRTGQISAWITDPNCKRAVSLILSKQMPDLADSIDL
VCQEKSWEGIIKKIWPRTKYVLATITGSMAQYIPALEFYTGGLPVVSSLYGSSEAFFGINMKPLCS
PYDVSYTFIPNMAYYEFLPIDNHQDPNCTNRKDAHLKDHIVDLANVKIGQHYELLVTTFTGLYR
YRMEDILLVTGFHNSTPQFKFVQRTNVVLSIHTDKTTEQDLQKAVAIAMQILQPLGFFLLEYSSY
ADTSSIPGHYVLFWELQLRPNDDTPELDQVKMEKCCSLVEQSLDQKYKWLRNQSFSTIGPLEIRV
VKQGTFNVLMDFYVSQGTSLNQYKTPKNIKSEKAIVILDSRVVGKFYSREVPNQDS*
>CcGH3.5
MLPTFDPRDTEAGSRILEDISSNAGHIQEQVLEEILTKNASTDYLKSFLNGHSDKGLFKNKVPVVD
YEDIKIYIDRIAVDGEPSRILTNESITELLISSGTSGGKQKWIPKTAEEGERRAFFFCLLATVQNRYL
HGLNDGKALFFVLINPDIHTPGGLVLRTTTASEIKNRKDRYPEVIFCEDTNQSLYSQLLCGLVQRD
AIVSIGTFFASGLQRIIKFFEEHWQEMSSNIRTGQMSDWITDPKCKRTVSLILSKQMPDLADSIDLV
CQEKSWEGIIKKIWPRTKYVQAIITGSMAQYIPALEFYTGRLPVVSPAYVSSEACFGINLKPLCSPY
DVSYTIIPNMAYYEFIPIGNHQDPDCTNSKDAHLKDHIVDLANVKIGQHYELVVTTCTGLYRYRM
GDVLLVTGFHNITPQFKFVQRRNVVLSIDTDKTTEQDLQNAVTIAMHILEPLGFFLLDYSSYADTS
SIPGHYVLFWELQLRSNDDFPVLDQVKMEKCCSLVEQSLDLQYKMLRNRSISTIGPLEIRVVKQG
TFNVLMDFYVSRGTSLNQYKTPKNIKSEKAIEILDSRVVGKFYSTEVPNQDS*
>CcGH3.6
MSSNIRTGQMSDWITDPNCKRAVSLILSKQMPDLADSIDLVCQEKSWEGIIKKIWPRTKYVMAIIT
GSMAQYIPALEFYMGGLPVVSPLYGSSEALFGINMKPLCSPYDVSYTFIPNMAYYEFLPIDNHQD
PNCTNRKDAHLKDHIVDLANVKVGQHYELLVTTFTGLYRYRMGDIVLVTGFHNSTPQFKFGQR
TNVVLSIHTDKTTEQDLQKAIATAIQILEPLGFFLLDYSSYADTSSIPGHYVLFWELQLRSNDDIPE
LDQVKMEKCCSLVEQSLDQEYKMLKNQSISTIGPLEIRVVKQGTFNVLMDFYLSQGTSLNQYKT
PKNIKSEKAIEILDSRVVGKFYSREVPNQDS*

>CcGH3.7
METDWNTKALEFIEEMTKNAGEVQKMVLADILKQNGETEYLQRFNLDGATDTKTFTSKVPCITY
EDIRPEIQRMENGDRSAILTALPVSDFLLSTATTSGKRKLIPMPEEEWNRRQVLSSLEMPVMNVY
VPDLNKGKGLYFYFPWPEIRTPGGQMILTALTKFYRSEHFKSQRLDPYTQYTSPYESVMCTDYV
QSMYVQLLCGLYQHKQINRVGASSASALLRVIKFFELNWQDLVHDIRVGSLNPKITYEPLRECM
AQVMKSDPELADFLTIECSRGNWEGIIQRIWPNAKYLQTIITGSMIQYAPLLVYYSGGLPIATAKY
ASSECCFGINLNPISKPEDVSFTFMPNLAYFEFIPQEHLSSKMDIVNIVITTHAGLYRYQMGDILRV
TGFHNSAPNFKFLRRKGVLLSIELDKTDEVELQMAMDNASQILQEYNVCLVDYTSHASKKMAP
GHYVIYWELSVRDSDKNAQSDEVISRCCPIIENSFSLIYKQHRVHGAIAALEIRVLKNGTFQDLVE
FAVSRGASIGQYKVPRCVETGAVLEFLDSRVVSSHFSPCLPSLASEESI*
>CcGH3.8
MSDWITDPNCKRAVSLILSKQMPDLADSIDLVCQEKSWEGIIKKIWPRTKYVLAIITGSMAQYIPA
LEFYTGGLPVVSPLYGSSEAFFGINMNPLCSPYDVSYTFIPNMAYYEFLPIDNHQDPNCTYRKDA
HLKDHILDLNNVKIGQHYELLVTTFTGLYRYRMGDILLVTGFHNSTPHFKFVQRTNVVLSIHTDK
TTEQDLQKAVAIAMQILEPLGFFLLDYSSYADTSSIPGHYVLFWELQLRSNDDIPELDQVKMEKC
CSLVEQSLDQKYKLLRNQSISTIGPLEIRVVKQGTFNVLMDFYVSQGTSLNQYKTPKNIKSEKVIE
ILDSRVVGKFYSREVPNQDS*
>CcGH3.9
MDGKKLEYKGEKALKELEKLTANAAEVQEEVLKMILTQNKGAEYLNKYMTGVESKSQVPHFK
RCVPVTTYKDVRPYIQRIANGDNSNLITSQPVTEMLCSSGTSAGEPKLMPSIEEDLDRRTFLYNLI
MPIINQYVGGLDEGKAMFLYFVKAEMSTPCGLPARTVLTSFYKSQHFKNRSHDPYNDFTSPDQTI
LCYDSNQSMYCQLLAGLVFRHQVLRLGAVFASAFLRAISFLERNWRKLCQDIRTGKLDHAMITD
AQCQSAMYSRVLLRPQPLVADEIESICSSKSWKGIVRRLWPKAKYIEAVITGSMSQYIPSLEYYSD
GKLPLVCTMYASSECYFGVNLKPFCKPADVSFTLLPNMGHFEFIPLGESATWSIDLDEEEEEEEEV
PPNKLVDLVHVRVGCYYELVVTTFAGLYRYRIGDVLQVTGFHNQAPQFRFICRRNVVLSVDND
KTNEEDLHKSITAAKKLLEPYNALLVEYTSCADASTVPGHYVIYWEIAYNNGLVDEAFAIDPTVL
QECCAAVEERLDYTYRRCRTLDKSVGPLEIRIVEAGTFESLMDFFINQGASINQYKTPRCIKSKAA
LKLLNSNVKASYFSSRDPCWNP*
>CcGH3.10
MAVDSVLSSPLGPPACEKDAKALRFIEEMTRNADVVQEKVLAEILTRNGQTEYLQRFKLGGFTD
RLTFKSEIPVITYEDLQPEIQRIANGDRSHILSAHPISEFLTSSGTSAGERKLMPTIKEELDRRQLLYS
LLMPVMNLYVPGLDKGKGLYFLFVKSETKTPGGLLARPVLTSYYKSDHFKTRPYDPYNVYTSPN
EAILCPDSFQSMYAQMLCGLYEREQVLRVGAVFASGLLRAIRFLNLNWKQLTHDIRTGSLNPRV
TDPLVRECT*
>CcGH3.11
MAYFEFLPHDPNSPGFTRDSPPKLVDLADVEIGKEYELVITTYAGLYRYRVGDILRVTGFHNSAP
QFRFVRRKNVLLSIDSDKTDEAELQKAVENASQLLREYNTSVVEYTSFADAKTIPGHYVIYWELL
VKDAAHSPSDEALNGCCLAMEESLNSVYRQGRVADNSIGPLEIRVVKSGTFEELMDYAISRGASI
NQYKVPRCVSFTPIVELLDSRVVSVHFSPSLPRWTPERRR*
>CcGH3.12
MKAMHSPCCSPEEVIFGPDFHQSLYCHLLCGLIQREEVQVISSTFAHSIVFAFRTFEQDWEELCTDI
REGVLSSRISVPSVRTAMSKLLKPNPELADLIHNKCQGLSNWYGLIPELFPNTRYIYGIMTGSMEP
YLKKLRHYAGELPLLSADYGSSEGWIGVNVNPKLPPEMATFAVLPNIGYFEFIPLRQSLDGLEAK
PVGLAEVKAGEEYEVIVTSFAGLYRYRLGDVVKVKGFHNSTPELQFICRRNLLLTINIDKNTEKD
LQLAVEAAAKVLAENRLEVVDFTSRIDSSTEPGHYVIFWEISGEASDEVLEECCNCLDKSFLDAG
YLSSRKVKAIGALELRIVKRGTFHKILDHYVGLGAAVSQFKTPRCVGPTNNKVLQILCNNVVKSY
SSTAF*
>CcGH3.13
MPEAPKVSSTNHQESCLDDKNQQVLQFIEDVTSNAKEVQRRVLNEILSRNAGVEYLQRHGFNGQ
IDYETFKSTFPVVTYDDLKPDVDRIANGDTSPILCSQPISECLTSSGTSGGERKLMPTIEEELGRRSF
LYSLLMPVMNQFVLDLDKGKGMYFLFVKSEAKTPGGLLARPVLTSYYKSSHFRDRPYDPYASY
TSPNEAILCSDSYQSMYSQMLCGLCQKDEVLRVGAVFASGFIRAIRFLQNHWPLLCNDIRTGTLN
PEIIDPSVREAVVKILKPNPQLAEVLEAECRKESWKGIIPRIWPNTKYIDVIVTGTMSQYIDTLNFY
GNNLPLVCTMYASSECYFGVNLNPLCMPSEVVYTLIPTMAYFEFLPVTSENEPVPLNPNKHHKLV
DLVDVQLGQDYELVITTYAGLYRYHVGDILRVAGFKNKAPQFTFICRKNVALSIDSDKTDEVEL
HNAVTKAANSHLLQFDASLIEYTSYADTSTIPGHYVLYWEIGFNSAKSIPESVFEDCCLTVEESLN
SVYRQGRVSDNSIGPLEIKVVKNGTFDKVMDFAISNGASINQYKAPRCVKYAPIVEILNSGVVFN
YFSPKCPKWSPGHKQWCTN*
>CcGH3.14

MERNGEYDIIGWFDEVAETADSVQTQTLSQILRLNYGVEYLKKWFRDINIQEMEENALESLFTSL
VPLASHADLEHYIQRIADGDDAPLLTQEAITNLSLSSGTTEGRQKFVPFTRHSSKTTLQIFKLAAA
YRSRIYPIREGGRILELIYSSKQFKTKGGLTVGTATTHYYASEEFKIKQEQTKSFTCSPEAVISSGD
YNQSTYCHLLLGLHFRDEVEFITSTFAYSIVQAFRSFEELWREICDDIREGSLSSRITITKVRNAVL
DIISPNPCLASRIASTCEELEEKDWFCMIPKLWPNAKYVVYSIMTGSMQPYLRKLRHYAGNLPLVS
ADYGSTESWIGVNVDPSSPPEKVTFAVMPNFSYFEFIPLHRQSQQNANSNTDDFIEEDPVPLSQVK
LGQEYEIVLTTFTGLYRYRLGDVVEVAGFHKKIPKLNFICRRKLILTVNIDKNTEKDLQLVVERGS
QALSKSKAELVDFTSHADAAKQPGHYVIYWEINGEVEERVLRECCREMDASFVDHGYVVSRRT
NSIGPLELCIVEKGTFKKILEHFIRNGAALSQFKIPRCTSNQVLLKILDASTIKRFRSTAYGQEL*
>CcGH3.15
MPEAPKDEGAYAHAHDTNTIYSVAEEKNKKFLQFIEEVTANADEVQKRVLAEILSRNAHVECLK
RHGLNGQTDRETFKKIMPVTTYEDIQPDVNRIANGDKSQIICSQPISEFLTSSGTSGGERKLMPTTE
EELERRSLLYNLLMPVMSQFVPGLERGKGMYFLFIKSEAKTPGGLVARPVLTSYYKSSHFKNRSF
DPYMNYTSPNETILCPDSYQSMFSQMLCGLCLNKEVLRVGAVFASGFIRAIRFLEKHWSLLCNDI
RTGTLNPRITDPSVREAVMKILKPDPQLAEFIEGECGMESWQGIITRLWPNTKYIDVIVTGTMSQY
IPTLDYYSNGLPLVCTMYASSECFFGVNLNPLCKPSEVSYTLIPTLGYFEFLPVDRNNGVTNCISM
PKSLNGKEKQELVDLADVKLGQEYELVVTTYAGLYRYRVGDVLRVAGFKNKAPQFNFICRKNV
VLSIDADKTDEVELQNAVKNAESHLVPFDAQVTDYTSYADTTTIPGHYVLFWELSLNGSTPIPPS
VFEDCCLAVEESLNSVYRQGRVSDKSIGPLEIKIVEAGTFDKLMDYALILGASINQYKTPRCVKFA
PIVELLNSRVKASYCSPKCPKWIAGHKQWNINVK*
>CcGH3.16
MAVDSTPLAPPACENDAKALQFIEDMTRNCDSVQEKVLAEILSRNAQTEYLRDFKLGGATDRDS
FKSKIPVVTYEDLHPYIQRIANGDRSPILSSHPISEFLTSSGTSAGERKLMPTIHEEWDRRQKLYSLL
MPVMNLYVPDLDKGKGLYFLFVKAEAKTPSGLVARPVLTGYYKSDKFKNRPYDPYLVYTSPDE
AILCADSFQSMYTQMLCGLLMREEVLRMGAVFASGLLRAIRFLQLNWKQLSEDIKTGVLNPKVT
DPSVRKRMAEILKPNSDLADFIVKECEGQNWDRIITRIWPNTKYLDVIVTGAMAQYIPTLDYYSV
GREYELVITTYAGLCRYRVGDILRVTGFHNSAPQFKFIRRKNVLLSIDADKTDEAELQKGIENASA
LLREFDTRVVEYTSYADTKIIPGHYVIYWELLVKDPANPPTHEVLNQCCLAIEEALNSVYRQGRV
ADNSIGPLEIRVVKNGTFEELMDYAISRGASINQYKVPRCVSFAPIMELLDSRVVSVHYSPAAPH
WAPERRR*
>CcGH3.17
MPLPALDSTDSEALLELLEKVTADASQIQDELLQEVLTTNANTQYLKGFLNGYSDKQLFKKAVP
VVEYQDIEPFVDRIANGESSHLISAHPITELLLSSGTSGGKRKAIPTTAEEPRRRAFYASLTATILNK
HIEFWNQGKQMNFRFIMPEMTTLGGLAVTNAVSSNFRRSRMQNRSNCWNNDTSPDEVILCQDIK
QSMYCQLLCGLVQRDAVVRIGTTYASGFLRVIKFLEEHWQELCSNIKTGHISDWIIDPGCQKAVS
SILSQQMPGLADSIEIECRSGSWGGIVKRLWPRTKCIEVVSTGTMTQYIPNLEFYCGGVPLVSMYY
AASEGFFGLNLKPLSDPYNVSYTLVPWMAYYDTLGDHDLKGELVDLVNVQIGQQYELVVTTFT
GLYRYRVGDVLMVTDFRNNTPQFKVVQRRNVVLSIDLDKTTEEGLLKAVSKAMQILEPLGCLLT
DYSSYADMSCFPGHYVLFWELQMRENAGIAAVLERVQMEGCCNVVEESLDGMYKTLRRRSNVI
DPLEIRVVKRGTFDGLMDLFLSEGASLNQYKTPKSIKSEKAIQFLNSMVVETFFSRVLPAFPPTG*
>Cc00_g28970_not selected for analysis
MRGWLTWPIGLKGLNHGVNLFLKTIMLPTFDPRDTEAGSRILEDITSNAGHIQEQVLEKILTKNAS
SDYLKGFLNGHSDKGLFKNKVPVVDYEDIKIYIDRIALDGEPSRILTNESITELLKSSGTSGGTQK
WIPKTAEEGERRAFFSCLCDTVLNRYLQGLSDGKALLFVLINPDIHTPGGLVLRTTSESEIKNRKD
RYPQFILCEDTNQSLYSQLLCGLVQRDAIASVGTYSPRVC*
>Cc02_g39040_not selected for analysis
MDLSAESFGVAPRRNMGMLEEMPLSLDPEDVIAEFEAMTRDAGRVQIETLKKILEENGRTEYLQ
RWGLDGRTDPESYKSCVPLVTHEDLEPYINRIVDGDDSSILTGNPITTISLSSGTTRGMPKFVPFND
ELMESTMQIYKTSYSFRNRQVLHFLASRKFDWFSGFPYMAFRSHIDLSCGSPCLNYV*
>Cc00_g04550_not selected for analysis
MRMKGWITWAMGLKELNHGVNLFLKTIMLPTFDPRDTEAGSRILEDITSNAGHIQEQVLEEILTK
NASTDYLKGFLNGQSDKGLFKNKVPVVDYEDIKIYIDRIALDGEPSRILTNESITELLKSSGTSGGK
QKWIPKTSEEGERRAFFSCLCDTVLNRYFQGLNDGKALLFVLINPDIATPGGLLLRTASESEIKNR
KDRFPQFILCEDTNQSLYSQLLCGLVQ*
