# Supplementary material for: Genome-wide analysis, transcription factor network approach and gene expression profile of GH3 genes over early somatic embryogenesis in Coffea spp
Source: BMC Genomics. 2019 Nov 6;20:812. doi: 10.1186/s12864-019-6176-1 (PMC6836404; doi:10.1186/s12864-019-6176-1)
Supplement: Supplementary file 2 — Additional file 2: Data S2. Complete set of amino acid sequences used into the network analysis. [file 12864_2019_6176_MOESM2_ESM.pdf]

Supplementary data 2. Complete set of amino acid sequences used into the network analysis.

>Cc04_g01870
MSNLTSASNEASVSSVNRNDNGSGGSIYPPQQYFAPPNQTQVAQPQQAVKKKRNLPGNPDPDAE
VLALSPKTLLATNRFICEICNKGFQRDQNLQLHRRGHNLPWKLKQRTNKEVKKKVYVCPEASCV
HHDPSRALGDLTGIKKHFCRKHGEKKWKCDKCSKRYAVQSDWKAHSKTCGTREYRCDCGTLF
SRRDSFITHRAFCDALAEESARAIPGNPLLSSQAAGSSTAPRHVNNINPLQQQFGNQDNPHLFSLK
KEQHTFNLRPEIPPWLACSPMPAGAGPGPGPPPVDLTSSIFPSPRLGPGFSECHQELSLHENPNPNL
GPNLPPFHPTTSPHISATALLQKAAQMGAAMGSSKAGAGGAAPPAVMLNTRPHQAHVSAAAAD
SATNITTGNFGLNLSSREDLASGTFVNGLASYGNKAVDPAAGGPPPPTSFLQDMMMNSLSSSSAT
TGFEGSAFEDAFGGILNPKKTSNSIVGGGNDRMTRDFLGLRPLSHSDIFNIAGFSNCMNTTASNEH
HQNASQKSWQG*
>Cc04_g16110
MSRCCSQCGSNGHNSHTCGGGSSSASAADGGGGSDSGSGSISEFMLFGVRVKVDPMRKSVSMN
NLSDYEHHPSNASTVQKNSVHLDASAPKAADDVAAGYASADDALPTHHSSAAARERKRGVPW
TEEEHKLFLLGLQKVGKGDWRGISRNFVKTRTPTQVASHAQKYYLRKSNLNRRRRRSSLFDLTP
DSVTGVPMEEGDGHLDETANPALPATSMPPNESSSVNGFAGAPFPVTVGPILVPVQVQNPLSSMT
ACQADAFSNGSALVPVPVPVIPVPNYATSAVFNLNQRVAAESVSLRLSLSSSDHQRQSSTRHRAF
QRMPSFKNGDGIITVA*
>Cc02_g15550
METRFANSPLINEQEDSENSPENSGDSPRFCMLNDSKMTCTSSPKKSRRSIQKRVVSVPIKDPEGS
RLKGEMGVPPSDSWAWRKYGQKPIKGSPYPRGYYRCSSSKGCPARKQVERSRVDPNMLVVTYS
CEHNHPWPTPRNQNHHNQNQNSSPSINKATKTTSTKGESTNSANSDEEEGEEEEKPAKFASQAEL
NSDEKFVPLISSASDYGWFSEFESTTSCTMLESPILTEARVTDADLAMAFTMRDDDESFFADLGEL
PECSTVFHRGMMEREEERRRHGLTPWCGTTG*
>Cc01_g19020
MDPLYPVKEEYPDQYHAGSSSSPPGTLAPQPMEGLHDAGPPPFLTKTFDMVDDSTTDHIVSWSR
GGHSFVVWDPHAFSTALLPRYFKHNNFSSFVRQLNTYGFRKIDPDKWEFANEAFLRGQKHLLRN
IRRRKAPSQPTSPQQALGPCVEVGRFGLDAEVDRLRRDKQVLMMELVKLRQQQQSTRAHLQSM
EVRLQGTEKKQQHMMSFLAKAMQNPEFIHQLIQQKDKRKELEEAFTKKRPRPIDQGPGGGESSR
SGEVRNHVKAEPSEFGDPYGYQVSELEALALEMQGFGRAARRDRDEEQDELDKELDEGFWEEL
LNEGFDEEGETGKEGGGEEDVNVLADRFGSLGSSPK*
>Cc07_g03350
MGRPPCCDKVGVKKGPWTPEEDIMLVTYVQEHGPGNWRAVPDNTGLRRCSKSCRLRWTNYLR
PGIKRGNFTDQEEKMIIQLQALLGNKWAAIAAYLPERTDNDIKNFWNTHLKKKLKKLQTGSDSS
SARNALSSSHSISKGQWERRLQTDIKTAKQALHDALSLEGSIPAPDSKPCDGHRSYTEPGQGSSTY
ASSTENIARLLKGWVKNPSPRKSEQSKSSSTQQSFCNAATDCTSSDGTPSAESKSGIDLSEAFESLF
GFESFESSTSEFSQSTSPEASIFQGESKPDQGGAQVPFSMLLENWLLDGKDDLTNFPFEETANLF*
>Cc09_g04020
MASMNNWLGFSLSPQEIRSQPHQDHSQNTVPRLDTFSSEELSGTDVSGDCFDLSSHASTIPSLNLP
APFGILEAFNRNNQSQDWSFKELNVNPNTSYKTAGSEMSMLMGRSCNNNNHNLENQEPKLENF
LGVGGQNSLHLPSQTVEASSSNGNGTIGLSMIKNWLRNNPSTSQPENKIINGNDGAVGCSNNAQ
NLSLSMSTGSQSTSALPLLTAASCSDGEGGGGESSTSDNTNTNKQQNGGIGITSLDGQSGAIEAVP
RKSIDTFGQRTSIYRGVTRHRWTGRYEAHLWDNSCRREGQTRKGRQGMVIYCLPLLVTISLLFIF
LLNLSLLVLTIFFFVSHFAYVLMGGGYNCFAVYLGGYDKEEKAARAYDLAALKYWGTTTTTNF
PISNYEKEIEEMKHMTRQEYVASLRRKSSGFSRGASIYRGVTRHHQHGRWQARIGRVAGNKDLY
LGTFSTQEEAAEAYDIAAIKFRGLNAVTNFEISRYDVKSILESSTLPIGGAAKRLKDAEQAEMALD
AHRTNNDNLSSHLTDGMSSYGAQHAWPTIAFQQAQPLTMHYPYGQQQRLWCKQENDSDVSHS
FHDLHQLQLGNTHNFLQPSVLHNLMSLDSSSMEHSSSCNSVIYSNGSNDNATYQGVGYGSSSSY
LLPMSTAVIAEEGSQNQGNGFGENGVKAIGYENMFGSSDPYQTRNFYYHPLPSSNGTLRAARTN
NMAVCHGSSTFTVGNDT*
>Cc04_g16910
MEGGDDHHLHHHHHRPNFPFQLLEKKEDESASCSSSGAAGFPSLAISSADNTSQNPSRSTSSSLQI
SAEPSKKAPPKRTSTKDRHTKVDGRGRRIRMPALCAARVFQLTRELGHKSDGETIEWLLQQAEP
AVIAATGTGTIPANFTSLNISLRSSGSSMSVPSQLRSSYFSPNFSMPPRRSLFQGIGLSTDNSPSTTTL
LNFQSGNINPSSMLQAKQELRENSTHNTLDLSDAAAAAAAGEESPLARKRRNDQELQHHQQQNI
GNYLLQSSTGAMPASHASIPANFWMVQANSSNQVMGGHDPIWTFPNVNNSAAAAAALYRGSM
SSGLHFMNFPTPVALLPSQQLGTSSIGPTGGSGGGGGNGGLAEAQLGMLSGLNPYRSSGGGVSES
PASGSHSQHHGDDRHDTTSHHS*
>Cc09_g03140

MMERVENEGVNVGIPVDKKPKKEAAISTSMKKITVGGSRKGCMRGKGGPENAFCTYRGVRQRT
WGKWVAEIREPNHGARVWLGTFNTSYEAARAYDDAAKRLYGKCAKLNLPEEDQPPSPPGSSSV
ASAAYSNNTGYKNGQDLTNEHQSPELEDKKNGTSVLDEVSIFKDINGEFAFDETPAPSLLGEEQIL
NWPEYPFDNGFHWSNDGGISVGGLIDHAVVYKLLGPPN*
>Cc07_g09170
MGTGEESTPAKPSKPTSTNQETPATPSYPDWSTSMQAYYGAGATPPFFASTVASPSPHPYLWGN
QHPLMPPYGTPVPYPALYPGGVYAHPNMAMAPGAVQVPIESDAKAPDGKDRNTNKKLKGPSG
NPGLIAVKAGESGKAASGSGNDGATQSAESGSEGSSDGSDENNNHELSATKKGSFDQMLADGA
TAQNNTSVAHFQNSVPGNPVVSVPATNLNIGMDLWNPSSGASGAMKMRPNPGVSPAVAPGMM
TDQWIQDERELKRQKRKQSNRESARRSRLRKQAECEELQQRVESLNSENRALRDELQKVSEECE
KLTSENNSIKEELTRLCGPEAVAKLESSSITQLETNGDEDDH*
>Cc04_g14150
MGLTSLQVCMDSSDWLQGTIHEETGMDSSSPSGDMLTCSRPLIERRLRPQHDQALNCPRCDSTH
TKFCYYNNYSLSQPRYFCKTCRRYWTKGGTLRNIPVGGGCRKNKKVSSKKSNDQHLHQPSATSP
TMNPTDLHLAFPDQMQFQHLSNILGNANGFMENKYNLMLENPTPIDFMENKYEALVGNSSRNY
DFMGNGDMGILGCEMSSPAGMISAPNFHNFCTAPFGNMSIDGNTPGTLMLPYEAHEDQNAMDV
KPNAKLLSLEWHDQQGCSDHGGKDSYGYYNGVGSSWPGLMNGYGTPTTNPLV*
>Cc02_g37070
MGSAQKLELHGDQSRNGSNLNNPWPRPPSTAVQPLRMPPAPPMAIPLISLKEEPDDHGEDKPPIP
MARSSQPLPMPTPLQMRQPVAPPTPPKRTSTKDRHTKVEGRGRRIRMPATCAARVFQLTKELGH
KSDGETIRWLLEHAEPAIIAATGTGTVPAIAMSVNGTLKVPTTSPAAADNSNTVKRKRKRPSNYS
ADQHVNMPTNTDDNVATSTGNTSTSSTTTMSTFSSNNPSILAPLMAIQPTNQSQILAIPPNVNVNV
TPFINVSARPISAFIASSSTTPSAANATRTQVLREFSLEIHDKHEQQN*
>Cc10_g04530
MALMLDNCEGILLSLDSHKSVPAPFLTKTYQLVDDPSTDHIVSWGEDDTTFVVWRPPEFARDLL
PNYFKHNNFSSFVRQLNTYGFRKIVPDRWEFANEFFKKGEKHLLCEIHRRKTAQPQVAINHHHH
HHPLNNPISSPTFFPNSISSSSVTALSEDNERLRRSNNMLMSELAHMRKLYNDIIYFVQNHVKPVA
PSNTFPSSLLPSSATPIIHNTTNSSSSLMQKPLNQLIGFHHHHYPKQNEPMDSSNISRTKLFGVPLHS
KKRLHPECSNSMVETNKARLVMEKDDLGLNLMPPSPC*
>Cc00_g28360
MAPPPVERNGGESTTTPPPPGDGPRSLPTPFLTKTYQLVDDRTIDDVISWNEDGSTFIVWNPTEFA
RDLLPKYFKHNNFSSFVRQLNTYGFRKVVPDRWEFSNDCFRRGEKGLLCDIQRRKLAAPPVAGA
TISPVTVPTPTAAVSAIPPPRTVSPTDSGEEQVVSSTSSPSGFRETATAGGSTAELIGENERLRKEN
MQLNKELSHMKSMCSNVYVLMSNYSNHSNGATANSKAEGSSSQALKALDLLPPKPIFDESGATT
ECGGEDRMAVDEAGARIFGVSIGVKRGRENGAAAAAEHDKELQLQQPGTADVKSEPIDDENSN
GDDQQTHWLRQCRIQNQRCVIK*
>Cc07_g05550
MPSADSSERRGTKANQVGSGAPPSEPEQLQCPRCESTNTKFCYYNNYNFSQPRHFCKSCRRYWT
HGGTLRDIPVGGGSRKNAKRSRTVASAAASSAFSFSSSTSTYEHRHTQATTSQLLTPLGVDHGGA
VPLISDAKSGVNACGSFTSLLNTQGPGFFALGGFGVGLGAGVEDMGFGLARAVWPFPGLGVGE
GGPTGVGGPSVLGNTWQLQSGEAGIVGGDCFTLSDVAISTAGHGMKRDVF*
>Cc08_g09710
METPEFFQGGYYNTHMAPEKRLSDAKNSDHFIIDDLLDYPNDDGMVADGTFDTTITAGTSTDSS
TVVDTSCNSSFSGSTEPHLPGGDMGCRNFTDGQFSSELCVPYDDLAELEWLSNFVEESFSSEDLQ
KLQIISGMKARTNEVSETHDDQPEPNRETATPMLRPEMSVPAKARSKRSRAAPCNWTSRLLMVT
PSTTTLTAATTTTIMAAAMSSSSDSEITPSTGKKAVKAPSKKKEVYDQNASMANGEGRKCLHCA
TDKTPQWRTGPMGPKTLCNACGVRYKSGRLVPEYRPAASPTFVLTKHSNSHRKVLELRRQKEM
LRAQQQQHQFLHQNMMFDVSSNGDDYLIHQHIGPDFRQLI*
>Cc07_g05080
MFSSGSEITHFAQLSPYLCPPSSTTSSSTSSTLGLNGNNEAFLLLHHQDLLSHYLAVAPMIETANT
MTAPTSTKKTTTASTTNPQISSATPLHTRYRRKQPAKRDRHSKICTAQGPRDRRVRLSMDIARKF
FGLQDLLGYDKASQTLDWLLTKSKAAIKELLDMKDTDHDANAKCLCFWSEAGNVVAAGTNTK
EDESKKTASKRKRKKTNQAKNPKMVALSKLVKESRAKARARARERTREKISLRKLSIDQKSPDLI
PLSPDHTKPAAWSQIGTCKTKDSSMLGTISSLKLNASIQEDDGFFCFNPRAGAIGAEEISQDTPRIIR
KPKPSSILGFQRNLILSKEASPNFDSWSLVTNGNWGISSSMTSSSLCAVTNTSSTKVLQICGKPSED
KSNNNQVPCGRQIHRST*
>Cc05_g07790
MSAGCYVNSDKPTPLPQKTRKEKSKEKLSCRLQLSLFTASHERIAFYLDRKHTLNSHLSSFTLLLI
PITGCHKHIKTQRILHFSASLVKVDIIAKRLNQALQKAKDSSEDDRGSNLKWFHPNSNRRSESSS
KEEEKPSRNTRQRILPFYIPFLFTFFFDPEAEVIALSPKTLMATNRFLCEICGKGFQRDQNLQLHRR

GHNLPWKLKQRTSKEVRKRVYYCPEKSCVHHHPSRALGDLTGIKKHFCRKHGEKKWKCEKCS
KRYAVQSDWKAHSKTCGTREYKCDCGTIFSRRDSFITHRAFCDALAEETARVTAASNVVAANSI
NYHFVGASLGPGMGQHFASMFKPISTNNETPANPIRQGLSLWMGGHGLSQTQESIGNNNLQEIH
HQLNPVVSTSGLVFTTDPFVKLTNNISSLPLSNVAKDGGSQVISVPSLFSTQHQSHQTHSTANMSA
TALLQKAAQMGATTTDTSFLGSFGLKCNDSQIQDGNRYCSPYGGTATSVATALQSSVTDHLSTL
NELQMYPSKRRHIQVDQDSTPTTGGGQTRDFLGVGIQSICHPSSINGWI*
>Cc06_g16660
MDGYRKTTLKPWKKGPARGKGGPQNAMCEYRGVRQRTWGKWVAEIREPKKRTRLWLGSFAT
AEEAAMAYDEAARRLYGPDAYVNLPHLRSNFNPLNKSQKFKWFPSNSLVSMFPSTGLLNLNAQ
PSVHVIHQRLQELKETGLLCQTSSASTSSSNPKSDVEYVDDHPHVESLEGKNNDEAEHPWENKSL
NQEEKPQIDLNEFLQQLGILKKEDQPGVSEVPSNFIDKDFSPKDDDVFTNLPENIFNWETPSELPGI
EDNQLVENSRFHVDNDNDDDPSFPPSIWNF*
>Cc02_g36000
MGRHSCCYKQKLRKGLWSPEEDEKLIKHINKYGHGCWSSVPKLAGLQRCGKSCRLRWINYLRP
DLKRGTFSLEEENLIIELHAVLGNRWSQIAAKLPGRTDNEIKNLWNSSIKKKLRQRGIDPNTHQPI
PEAENEEKGSATSKNNEKTSEGSNDQLNYGEAAAESSEQKMAPPDLQDYFSFQQLNYGSSTNIG
LSMNPNASSFFNLTTSRSAEMVSDQFNSAMSNSIMSAPSARMKPSISLPSENNSLGNFNVNKFPS
WDACAISANNGSTSNASSSSIELQSNCSFFDNNGFSWGAADCGKAEKDVVHMHSSVGDTEDIK
WTEYLQAPFLLSNSAAATIQNQTAQDIYSAETKSGISFTAPEGSVSTTTSWLQNHHHQQQPTIQA
VAELYNKHFQRLPAAFGQFS*
>Cc04_g00720
MLLRFLGDFFYIKVFAFSMASVENSQNLEPDDDEVNNDAHDLLLGDPHHYTITAVGPAFEQSPR
VAAAVDPTPSPPPPPPPFLQSSDIVPMLKEEPADNDVEGSIPVGLTPIHHLDKPMLLAPPKRSSKDR
HTKVEGRGRRIRMPATCAARIFQLTRELGHKSDGETIRWLLERAEPAIIEATGTGTVPAIAVSVNG
ALKIPTTSSTSMTATEGDGARKRRKRASTSEFYEVNDNSSFAPVAPITPQGLVPVWTVGAHGAPG
MVPTSVVPSGAFFMIPPTGATIAAAAAAAGAVPPRPSNQPQLWAIPAAAATPVFGVSGRPISNFV
ATGSPTGATSAQVLRDFSLEVYDKRELQFMVGAAAGAGNDQTPSSKS*
>Cc11_g17270
MAENKPTDINKDFRILASSDNKDDGGKKQLVPKRSSNKDRHKKVDGRGRRIRMPALCAARIFQL
TRELGHKTDGETIQWLLQQAEPSIIAATGTGTIPASALATAASGSSVSEQGNSVATGLHHPKFDEV
GPGMGIRMNWAALGGNIGRSPHVPAAAWPSVAAGFGSGFLHNSTLLSSNSVNQNLNSGPKFGF
QGLEFPNSAGFGQQLPGLELGLSQDGHMGSLNFQALQQFYQQIGQSGDGAGSGSANNQQQHHP
PPAAGGDDSQGSRQ*
>Cc02_g16110
MSCQSDPSRETSPQRKLGRGKIEIKRIENTTNRQVTFCKRRNGLLKKAYELSVLCDAEVALIVFSN
RGRLYEYANNSVKETIKRYKTVNSDSANTGSISEANAQHYQQEASKLRAQISNLQNSNRNMLGE
SLGSLNLRELKNIESKVERGISRVRSKKNELLFAEIEFMQKREVDLHNNNQYLRSKIAETERAQH
DMNLMPGSSDYELVSAQPFDARTFLQVNGLQLNNHYPRQEQRPLQLVYDFLTSLFTTHQLTYM
LRPLIMHL*
>Cc02_g04800
MVKASTLRKCSHCGHNGNYSRTCNGKGIKLFGVKINVVEDDYANRQNESIRKSKSMGNLETCN
GECNVPDDADGYVSDGLIHESSGAKTARERRKGKPWSEEEHRSFLLGLEKLGKGDWKGISKNFV
HSRTPTQVASHAQKYFLRLMTATERKKRRSSVFDIPLDEMVGLHPVPEFSSFPPLFFFFFFLGLLG
WGYVTIIIHTIDR*
>Cc02_g05970
MALLDQVSSVASVPMDYSRKRKSRRRDGTNNVADTLAKWKEYNDKLDSLDEEGKVVRKVPA
KGSKKGCMKGKGGPENARCNYRGVRQRTWGKWVAEIREPNRGSRLWLGTFGTAVEAALAYD
EAARSMYGPCARLNLPNYGPQQEASIESSSFPATSASDSTTTSGLSEVCPPADRDAEPNSNVKEED
GEGESRIQDTRPLTWVGAGSPMCTVKEEPKDGHMEALDMSIQAEAPVKNETFNSSDEPLDTLAW
DEMFDIDEMLSTLNSGPTHGSGSQYELVGINGGQFLNGNMQPSEFLYQMQNPDAKVLGSCQNM
EQTSQGVDYGFDFLEPGREENSQFALDDLGFFDLDADLGI*
>Cc02_g14960
MAESGGGIKLFGATIAVQVRQAKDEGNKGEEQQTVEKRPDKIIPCPRCKSMETKFCYFNNYNVN
QPRHFCKNCQRYWTAGGALRNVPVGAGRRKSKPPGRGFVAGLSDGCSLFDDASGVVHQFEFDH
HHHGVVEEWHVAAEHGDFQHIFPAKRRRSSTSSNSQPRSSSTLSCS*
>Cc10_g04630
MSDPYGTDDRSSFEPDSEPGGPPEPEDMNVFLHNLFQNSPATAGSSSLYRGSASMINSSSAASFDF
SDPGGFFAGEVKGRLEKTFSLAAAADCDAVTSSMDRGEFSGANKGLEASDAAINQAQPRSTKRS
RSAEVHNLSEKRRRSRINEKLKALQNLIPNSNKTDKASMLDEAIEYLKQLQLQVQMLTIRNGLSF

HPNYVSGSLQPMQLPMDFNEGDVMPNTSGGKHTLSSNQEVAMQSAFGVSNPKISSQQLAIASMT
NNSSSAFSFGLQSSAQNNPGVPNYLASAKDLCREDTLMQFPLDISRSGNNSSSGVSS*
>Cc04_g10620
MASEASSTPVASGNIEVFSWLKTLPVAPEYHPTLAEFQDPIAYIFKIEKEASQYGICKIVPPVPAPH
KKTAVSNLNKSLVARSGSPTFTTRQQQIGFCPRKQRPVQKPVWQSGENYTLEEFEAKAKAFERN
YLKKSRKKCLIPLEIETLYWKAIVDKPFSVEYANDMPGSAFAPRRGGKEGGGEGSNVNANVTVG
DTEWNMRGVSRAKGSLLTFMKEEIPGVTSPMVYVAMMFSWFAWHVEDHDLHSLNYLHTGAG
KTWYGVPWDAAAAFEEVIRVHGYGGEINPLVTFSTLGEKTTVMSPEVLIDAGVPCCRLVQNAGE
FVVTFPRAYHSGFSHGFNCGEAANIATPGWLTVAKDAAIRRASINCPPMVSHFQLLYDLALSSCS
RVPRGVRMEPRSSRLKDKKKGEGEMLVKDLFVQDVMQNNDLLYMLAEGSSVVILPQNSVVSSF
SSNSKAGSQSQVQPGLFPSLGSPDLMMKTTKSLLSEGIVQERKRGVLQGTGSCSMKETVSPSCFD
KRVPCSVRGNEFSALASESKNMETEKGRASRGDRLSEQGLFSCVTCGILCFACVAIVQPTDAAAT
YLITADRSEFKDWGETSDVSTVVNGDEVLPKSDSCSGWMYKRNPDELFDVPVQSGGLYQSVDD
EIVGLIPNTEAQKDTSSLGLLALTYGNSSDSDEDDVDANNHTEACQNEAKDCSPESGLYCHDAG
LHKGGSRNDVFSCSEFSCADVVPLQIIGSSDKQGTTKSTSESRRHPPPDGTIEYKRRSFPLMEIDNL
ADRCRHQVKEQDASSPSPLAHKAETIASTAIVEFENKTLPFAGRPDEDSSRMHVFCLQHAVQVEK
QLRSIGGVNVLLLCHPDYPNVEAQAKKMAEELGGHYVWSNISFRQASKEDEETIQAALESQEAI
HGNGDWAVKLGINLYYSASLSRSPLYSKQMPYNLVIYNAFGRSSPVNSPTKDDSLGKGPGKPKK
TVVAGKWCGKIWMSNQVHPFLAERDEEEQERGIPSCMKADLKPDRPLESTRVQTGETTARTCRT
GRKRKAAAEIRPAVKAKSAKVEERDKAAEDSPVNHSQHQCKSNRRNTQRKKENLESSNKGNKV
VRNRKQFNLETEEEQEGGPSTRLRKRTEKPSKGQGAKSLETKSVAKKQPNGLKAKKSPAGSNK
MKGKDEKTEYPCDMEGCTMGFGSKQELVLHKRNICPVKGCGKKFFSHKYLVQHRRVHVDERP
LKCPWKGCKMSFKWAWARTEHIRVHTGARPYVCAEPGCNQTFRFVSDFSRHKRKTGHSSSKKG
S*
>Cc10_g09120
MSSSDEGGSSCSTSQKRFRGIRLRKWGKWVSEIRVPGTQDRLWLGSYAAPEAAAMAHDVAYYC
LRENASLDDFNFPLMLPAGVQRGMSPRSVQKAATDAGMAVDAQIIAKHIPSWDGKEFSRELGD
QALNISVDDYL*
>Cc11_g05650
MDDDGLNMRNWGYYEPSSLKGNLALQLMSSVADRDTKPFLSGRDSGVMGAANGVYHPRDYLI
SGAPDHMDYVRDSWINHRDKFVHMFPGNPYNTVLPETSGTHQMQMLQQPEPSKDARVSVEDV
GVRKEPGPAKKRAAVSVPKTPKSKKPKKNPAVPKENGSSSGQRSKAVKKSMDVVINGIDLDISGI
PIPVCSCTGTPQQCYRWGCGGWQSACCTTTISMYPLPMSTKRRGARIAGRKMSQGAFKKVLEKL
AAEGYNFANPIDLRSHWAKHGTNKFVTIR*
>Cc04_g02150
MQDPSIYSQMKPQFPEQEHLKCPRCDSANTKFCYYNNYNLSQPRHFCKNCRRYWTKGGALRNIP
VGGGSRKNTKRSSSTTKRPSSASSAAPSTSPSSAAAVSSSSSPPSTTPQTLPKTEPYGLAPQALQPG
FDQDRRMILDVGGNGSFSSLLSSNGGQFGNFLEGLNPNASNLQLCGFGDHGPNQGPGQGHHGDP
HAGLQNGSNSEEGFLSNQGNGDSSCWNGSNGWPDLAIYTPGSNFQ*
>Cc08_g13970
MSSTSSSGSPSRPSGGKHPMYRGIRSRSGKWVSEIREPRKTTRIWLGTYPTPEMAAAAYDVAAIA
LKGSDIALNFPDRVSSYPAPASPSPSDIRSAAAGAAEMMKEKTAGGGDDDGKFPHDQNQPGSSD
HQELAFDQDRGLAGEDEFIDVEALIDMPNLLVDMAEGMLVSPPRIHSPPSSDSPEHSDVDSLWSY
F*
>Cc07_g09220
MNDSTASGDASASSSGNQAAAPPTSSAKKKRNLPGMPDPDAEVIALSPKTLLATNRFVCEICNKG
FQRDQNLQLHRRGHNLPWKLRQRSSKEVKKRVYVCPELTCVHHDPSRALGDLTGIKKHFCRKH
GEKKWKCDKCSKKYAVQSDWKAHSKICGTREYKCDCGTLFSRRDSFITHRAFCDALAEESAKA
QTVEALPAPTADDEEPKLQTLASSSPPPSVAAQPPPPAALVSSALPVQNPEGPENPKQSPAPPQILE
ETSVVTSLTGSCGSSSSSSSHGSTSSSVFASLFASSTTSGSLQSQTPEFTDLFRAVARPENASEVVAS
SSTEPISLCLATSHGSSIFGTAGQERRQYAPAPQPAMSATALLQKAAQIGAAATNASLLRGFGIVS
SASASSGQQEWSGRGIESDSASLAAGLGLGLPCDGGSSLKELMLGTPSVFGPKHTTLDLLGLGM
AAGGGPSGGFSALMTSIGGGLDVAAAAAATFGGSGEYSGKDMGRGS*
>Cc00_g13890
MASSGGSLNTSLNSYSSYSFPNQFMATSFSDLINGNNTSTSAGTTPAEIPKFKSFPPTSLPLSPPPVS
PSSFLAFPPSLSPSVLLDSPVLFSNSSTLPSPTTGTFSSLVRKDEDTKFVSDFSFQSQTRPPLSSSSLF
HASAGKSSSQEASMKQQAGGWNFDNKPSQFIESSAVKHGIRSELAPTTQRLGAEVPATQGSMQS
DPAPDSSHVHYSQPSQYVREQRKSDDGYNWRKYGQKQVKGSENPRSYYKCTFPNCPTKKKVER
NLEGHITEIVYKGNHNHPKPQSTRRSSSQSIQNPSYTHSEISHHSNTLGENGQTDSLPNAENSSASF
GDDEFDQTSAISNSRDDDENEPDAKRWKGENDSEAISVSGSKTVREPRIVVQTTSDIDILDDGYR

WRKYGQKVVKGNPNPRSYYKCTYSGCPVRKHVERASHDLRAVITTYEGKHNHDVPAARGSGS
YAVNRPSAINNNNDLPAAIKPLATNSNSVYRTNFANSLQNPIPRPQSQAPFTLQMLPSQGSFGISG
NSASANQVQQTGNAFHMAKQEPKDDLFFNSFLN*
>Cc06_g04990
MDWNGNLRPPFISRPTDTSLGFFYNYNYDPYQVIEMKHALQAPHAAVVSSMDKTIGYGNQDQK
KKRLTTDQLESLESSFQEEIKLDPDRKMKLAKELGLQPRQIAVWFQNRRARWKAKQLERLYDA
VKQELDVVSREKQKLQEEVVALRAILKEQVGKKPVSTGYTEMSGEETVESTSIPSSSKPRGVGGN
NLQTAECSYVFNVDDYNPVMPPYWAGLPSYP*
>Cc02_g35910
MPLSEFLRMARGKLESAQPKTASPADLSPATENDLVELLWENGQILMQGQSNRVKKSPNLNDFP
SQEPGIRDRFTGNVSTSKVGKFVEIGSTLNDAMPSVRSGETYLNQEDEMGPWLNYPVDDGFRSD
FCSEILPEISGVTGNEPSTLNSFGSVVKGSCNQVPQHSYTVPVHNGLDIEARNASKVSSSRTGLLSP
YSSQQCQLSVAPAGSGVSSVVMNTTSNNPVTFFGDTVQGQASPGGLVSMKMQNQKVASNFLNF
THFSRPAALVRANLEKTDGIAASCSSGIEKVSAVSSTSPVKSTHKPSSNSQKDICVHSQPKLVSTK
VDSRPSFDKPPEESCRAQRPDNLHWDDSIKNDKSSSPIISSSITKEVKDCENPVEPVVAASSVCSAN
SGEGASNDQMHTLKRKHCDNEESESRSEDIEEESVGIKKVAHARGVSGSKRSRAAEVHNLSERR
RRDRINEKMRALQELIPNCNKADKASMLDEAIEYLKTLQLQVQVMSMGAGLCMPPMMFPTGLQ
HMHPAHVPHFPPMAGGMGLGMGYGMGMLDLNSGSPRFPIFPVPPMQGAHFPSPTISGSSGFQGL
AGSNLQVFGHPGQGVPMSIPRAPLVPLVGQAPISSAVGVDTSRMGIHVEASNASPTLNSVVEVQN
KNSQLNHKTDTGSSISQTSSQATSKEFDQSAMTSKDDQAPDVCAAASVNMASTTDLVCDKEKGS
NGMCKE*
>Cc03_g01210
MNSQTPTPERVSSSSDGTQIKTCTDCGTTKTPLWRGGPAGPKSLCNACGIRSRKKRRALLGLNKE
EKKPKKSTSSSSSNNNNSSSTSNNYSSGSGDSNSSSGFSLKRKLLSFGRDVVVPLQRPRSSTNGQR
RKLGEVEQAAFLLMALSCGSVYA*
>Cc10_g13750
MGRGKIEIKRIENTNNRHVTYSKRKTGIMKKAKEITVLCDAKVSLIIFGTSGKMHEYISPSTNLVE
MLDAYQRSTGKKLWDAKHENLSNEIDRVKKENDSMQIELRHLKGEDITSLNYKELMSEIIKMIR
KTGEMLEEENKQLQYIWHQQEMANMKGAIGERDDVYQRVRDYPSQMPFAFRVQPMQPNLHER
I*
>Cc08_g16780
MFQRGTAPPACPIPSSWTAQHIPADEAELELHGMSGSSTTSKTISKRRFNDDQVRYLESMFEAESR
PELRVKQQLANKLGLQPRQVAIWFQNKRARSKSKQIEQDYSVLKASYDDLASKFESLKKENESL
HVEVQKLRQLTIRSGKEEYGREDIELEADQTTEFLLKASSSHEQSSTICDENFTRSIDYLVEATNSL
YMAQLADGSLTSTEDGCSFEANDLIDNSSCNSQLWEL*
>Cc03_g05520
MVRAPCCEKMGLKKGPWTPEEDQVLVSHIQKNGHGNWRALPKQAGLLRCGKSCRLRWTNYLR
PDIKRGNFSKEEEDTIIKLHETLGNRWSAIAARLPGRTDNEIKNVWHTHLKKRLKNHQATSQDIK
KQSTQVQRSDPRTPVKIDDQPDESNFTDDHHHRDGKVTTSLGCAPVSPQHSSSELSTVTENDSAA
IKQEKMDSPEYFPQIDESFWSEEFPVDNANVQSGIHDGLATNDMQFQFPLSPVAASDEDVNAYIS
NFDDGMDFWYDLFIRAGELPDLPEF*
>Cc07_g09150
MTSSMSHQADQQMVLISQYYPGIYSQLAPAEQGEVKSRRRRKKNRGGESSSSGVRKRKLSQEQV
NLLEINFGSEHKLESERKDRLASELGLDPRQVAVWFQNRRARWKSKKLEEEYSKLKSEHENTVV
EKCRLETEVLKLKEQVSEAEKEIQRLLERSDGVSSNSPSSSFSMEAIEPPFLGEFGMEGFENVFYVP
ETNYYVNGLEWVNIYNM*
>Cc00_g08780
MDPDERLRTNPWTKSSSSASDALRQLSPDNQSFTNFSSQQQKWEDSSSMDYRTRIEQQFSEFTNK
RMRETSNSNNVQDWDPRGMLNNLSFLEQKIHQLQDLVHLIVGRRCQAVGQSNELLVQQQQLIT
ADLTSIIVQLISTAGSLLPSVKHSLSSVNPPVTQLGQFGGSITPPVTSFDNSGRVVHCDVKKVEDQP
NEVDLIGNAGIEQNYVVEEHESKDEEDADEGENLPPGSYEILQLEKEEILAPHTHFCTICGKGFKR
DANLRMHMRGHGDEYKTPAALAKPNKESTSEPTLIKRITIRELTATRATLVAGATQRNSQS*
>Cc05_g07590
MAMKEKSKDVKPSTGKANAVKEVHFRGVRKRPWGRYAAEIRDPSKKSRVWLGTFDTAEEAAR
AYDAAAREFRGPKAKTNFPSPSEIQSPSNSSTVESSSGDGSTHAPVELDLTRRLGVAAGGGGAGS
EIPILHHPQAMAVLPNGQPVVFFQPMALNRADYPYRFGPVAMDYANVVGRGRGAVHSDSDSSS
VVDDNISDGDNMKRGGGLDLDLNFPPPVEA*
>Cc05_g07710
MESFENGGGDDFPDFSCGNLLDSIDFDDLFVGINDEDVLPDLEMDPEILAEFSLSGGEESEITTTST
SNEKMEENSAKEEVDKANYSGPDASSGSTLTQTDDMVTSKRDESVAANPSAKEADKARKSSTQ

SKANPQGKRKVKVDWTPELHRRFVQAVEQLGVDKAVPSRILEIMGIDCLTRHNIASHLQKYRSH
RKHLLAREAEAANWTQRRQMYGGAAAPAAAGGKREMNHWIAPTMGFPPMAPMPHFRPLHVW
GHPSVDQSLMPVWPKHLGPSPPPPPVTWAPAAPAHPPTPPPDPCFWHSHHARVPNSLTPGTPCFP
PHLAPTRFPTPPVPGIPPPALYKVDPGIAVPTAPAGQPVPQPPIDFHPSKESIDAAIGDVLSKPWQPL
PLGLKPPSVDSVLVELHRQGISRIPPTACV*
>Cc06_g11200
MGRKCSHCGNIGHNSRTCTAYSSGNISVMAAGLRLFGVQLDVSCNSAPSSSNSNSIAMKKSFSLD
CLSSSPTSSPSPSSSLSSSRISIGENSDKISIGYLSDGLLGRPQERKKGVPWTEEEHRSFLVGLEKLG
KGDWRGISRNFVTTRTPTQVASHAQKYFLRQASLNKKKRRSSLFDMVGSKNAIVRHQVFDLSFE
LNNDTTSANCLSSKASSDLDTAAHLLDLNSFAVDATTNHQDCIEVQEGSSSHHMDIDSMSIWPA
ASGSSSYQLSNISSITSTTGAPDLELTLAAPKTAAQNNPPPPPASILIMGPISVI*
>Cc10_g10960
MVKTEHKIQSEPAKPTKPIPSSSSSSSSSSSSKSKSSCKKKYKGVRMRSWGSWVSEIRAPNQKTRI
WLGSYSTAEAAARAYDAALLCLKGSSANLNFPISSYSLRFPESLVMSPKSIQRVAAAAAATVSTT
TTATATDPESPDSPSQPSTSSYSASESASSPTPSLSSSPPDHPPYIIEEEDISASVSLPPANLLDEAVM
PMTMNAAAGQWYTFDSPKYTDMVNGVFFDPMMMIEDVYEEGDINLWSFC*
>Cc01_g17430
MNHCVPDFEMDDDYGIPTSSSINASTTARPKKPAIGEEEIMELLWQNGQVVFQSQNQRSFKKSPN
RGGEPDQLHQSGASREIPSILENDNLSAAAPPQHQQQQLFMQEDEMASWLHYPLDDSSFDRDLY
SDLLYPPPPSASTTTTMTPTPSVPPRELRTTIVEIRPPPAVIPPRPPVPPPSKGTYPIEAAAPRLQNFM
HFSRLPKSRNESGYTRELTVVDSNETQTTVPESRVPEGDVRCGAVSCTAAAATSAAARDLATTA
CELTVTSSPGASGGSVSATAEPHHRPPTSIHKPSTSGVEDRKRKGRETEETDCPSEDIEFESADAKR
QARGSTSTKRSRAAEVHNLSERRRRDRINEKMRALQELIPRCNKSDKASMLDEAIEYLKSLQLQV
QLMSMGCGMLPMMYPSVQQYMPAMGMGMGMGMGMGMDLGMNRPVMPYPSFIPGSGMPSP
AVAANMGPRFPMPAFHMQPVPVPDSSRIQASNQLDPMLNSLVTPNPNQPRVPNFADPYQQFIGL
HQAQIPLPQAKSSGSTAQQAE*
>Cc05_g11430
MGENFHHHHQLQQQQQQPTTRQSSSRLSGLRNSTGAGEIVEVQGGHIVRSTGRKDRHSKVCTA
KGPRDRRVRLSAHTAIQFYDVQDRLGYDRPSKAVDWLIKKAKSAIDELAELPAWHPTTGSAAA
NTSFEQDQQAQKSRADNLQNQQQQHLGGLHQTDSAANPSGNSSSFLPPSLDSDSIADTIKSFFPM
GASAEANSSAMQFQSFPPPDLLSRTSSHPQDLQLSLQSFQDPILLHHHNQQQAQHHQNHPTSQHP
EQAEALFSGNTQLGGYDAAAWSEHHQQPAELGRNCSAKTSFFLRGDPFSPLIHLQFVLGWIHQL
YPSPLQIITFHTIRQCCQFIHHRYLALDLPLE*
>Cc07_g03470
MSEAKDPAIKLFGKTIQLPEAPAAAAASECGGDASALSNDISPDDTLVQDRPSSPNSLPEDSNLDR
SGAEEESDKDFSGEKGNDSKTEDGAQSMMPEELIDPTLSSLTKESSKAPSVDSEAAPAKASKAED
EQNETSNSQDKTLKKPDKILPCPRCNSMDTKFCYYNNYNVNQPRHFCKNCQRYWTAGGTMRN
VPVGAGRRKNKNSVSQYRHISVSETLHNSQPDLPNGIHHPTLKPNGTVLTFGSDAPLCESMASVL
NIAEKTMQNCTRNGFHRPEELRIPVPYGTGGNGDEHLSGSSVTTLSSKDEAAKDSLPDKQMPNC
QSISPQVPCFPGAPWPPYPWNAVPWGSSVPPAAFCPPGFPMQFYPAAPYWGCTVPGTWTVPWLS
PPPTSQNLNSRTSGPNSPTLGKHSRDDSIMKPVDSKKEEPQKESNPEKCLWVPKTLRIDDPGEAA
RSSIWATLGIKIDKVDSVGGAGLFKAFQPKGDEKTRVSETSTVLQANPAALSRSLSFHETS*
>Cc11_g12210
MVDNVYFFGTATTSINDDRDTSILSEFGWNLPPPPPPHSGTSAGLLHSGFDRIITDSDLAGNDTHT
TLFARENENTTAEPQPMTMSSSSSDDLPEKSTASGGSSADRQPSDTASKAKKKGQKRIRQPRFAF
MTKSEVDHLEDGYRWRKYGQKAVKNSPFPRSYYRCTNSKCTVKKRVERSSEDPTIVITTYEGQH
CHHTVGFPRGGLISHEVPFGSQLTPSSSQFYYPGVQFPQGSVGIQESFNVSRESGHSHAPSDPSGRI
FTDEGLLGDMVPPGMRKS*
>Cc00_g02380
MGSQGGVGKSTVGGTQIHEPKSSSLARQGSLYSLTLDEVQHHLGDLGKPLSSMNLDELLKTVWT
AEANQGTGGVDYGVHQHGQLPLALNRQSSLTLSRDLSKKTVDEVWQDIQQGQEHNHERKAQE
RQITLGEITLEDFLVKAGVVAEPTPSKNSGSSLGVDAVVLPQQNVPPQSQWTQYQIPSIHQSPPPQ
QQQQQNIMPVFMPGHPVQPQLTIGSNPMMDSAYPETQMTMSPSTLMGTLSDTQTPGRRRVGSG
DVIEKTVERRQKRMIKNRESAARSRARKQAYTHELENKVTRLEEENERLKRQREMEKALTTVPP
PEPKYQLRRTSSGPV*
>Cc02_g05280
MGNTEIPPEDGYTWRKYGQKDILNSRFPRSYFRCTHQKLYQCPAKKQVQRLDDDPNIFEVTYRG
DHTCHLSTVNMGGGSSTSGGAAAGPSTSRYGRDVDYPVADMADVMFNSGSSSSTSMDLIFSSM
DDKWPDSADKKN*
>Cc10_g07850

MEEYNHHLGENTGPRGSFLYGGPVLAAPSSSQSEGQDHPIVKTEAGASSHHQHGQHKFQYPSIIR
SHQTVQDHHHQPHHQESEISGEVEAIKAKIIAHPQYSNLLEAYMDCQKVGAPPEVVARLTAARQ
EFEARQRASLVTGRDVNKDPELDQFMEAYYDMLVKYREELTRPLQEAMEFMRRVETQLNMLG
NGPVRIFNSDEKCEGVGSSEEDQDNSGGETELPEIDPRAEDRELKNHLLRKYSGYLSSLKQELSK
KKKKGKLPKDARQKLLSWWELHYKWPYPSETEKVALAESTGLDQKQINNWFINQRKRHWKPS
EDMQFMVMDGLHAQNTALYMEGHYMGEGPYRLGP*
>Cc02_g31850
MFDGVPADQFHQFLAASSRTSLPIPLSFPLHHHGVSIPSSAASVSLASPPLPPTSATPPAPAFLGCFD
PYSSPLTLDVQVQPHHHHQQSGLHHQLHHQSPPPTSKNGEEKEEREGSISAAIPLDPWSNDEVLA
LLRIRSSMENWFPEITWEHVSRKLTGLGYHRSAEKCKEKFEEESRHFNSMNYNKNYRFFSDLDEL
YNDENPQVSTEKSQDLVKEKDKQEGDNKMDASTLQEEEDTGNTVAVANPSDQENAELVKESTK
SRKRKRNHKFEMFKGFCEAVVKKIVEQQEVLHNKLIEDMVRRDRESIARDEAWKCQEMDRINK
EIEMRAQEQAIACDRQGKIIDLLKKFTSGSEADQSLVRRIEDLLKVTNSSNSVTSSSEILPPSSLNSD
QTKLEAVTSASMAISHQNPTLKMVSAPNERGERIAGKRWPRDEVQALINLKCRLTNNSTSDDSIK
EGAKGPLWERISQGMLELGYKRSSKRCKEKWENINKYFRKTKDNNKKRSLDSRTCPYFHQLSTL
YGQGTLVAPSNLPENHQTSPENH*
>Cc09_g04320
MASSSNSPCAACKFLRRKCQPECVFAPYFPPDQPQKFANVHRVFGASNVTKLLNELQPHQREDA
VNSLAYEADMRLRDPVYGCVGVISLLQHQLRQLQMDLSCAKSELSKYQSLGITSHGLIAAAAAA
ATATTHHHHHPQNLGINFIGGGGSGREQYYHHQFFPRDQQQVIRAFDGGNNYDASSLLAMNVS
ASIGQLSQFQHPRAAGGDGRRTPIEPS*
>Cc02_g10630
MFSKTSTSLAPGFRFHPTDEELVRYYLRRKLCGKPFRLDAISEIDIYKAEPWELPGKSRLKTRDLE
WYFFSVLDKKYGNGSRTNRATEEGYWKTTGKDRPVRHKSQVVGMKKTLVYHSGRAPKGQRT
NWVMHEYRLIDEELEKAGIVQDAFVLCRVFQKSGSGPKNGEQYGAPFVEEEWEEDELEMVPKE
EAAEEAEVGDDSYWDGIDLDQILGTEISPDNDHMSLNVVAGENVASVEENTDSSNDPQKLLVSN
LPLEFYSGENIRGGEETTGSNNDSQNHLVGAGEYNCVPEQSDDQNSYNLPVHYDLHQKAVKRE
YIGEPSNALDAENVNYLLDEPYMDALDNLQFDDGAFLETNDLSNPIAADTSAFGMLEEYLNFYD
ADGEIPPYMNHDLPEMMGINETLVSGQASILPKESEEKAQSAEESSEKLLGRGDVASSSKEEPTKE
YFQYPFIKQASRMLGSIPAPPAFASEFPSEDATLLLDPSSSSTAHVTAGMIQIGNIGPGSHGMDWLF
GKHGRYNVVLSFGLSRGDDNSTTLESVVSIRPGKAAASGMSRGWFCFIFFWVLILSVGFKIGTCIC
AR*
>Cc02_g23810
MAIAATMSQENNNKDEHEHDHDMVMPGFRFHPTEEELIEFYLRRKVEGKRFNVELITFLDLYRY
DPWELPALAAIGEKEWFFYVPRDRKYRNGDRPNRVTTSGYWKATGADRMIRTENFRSIGLKKTL
VFYSGKAPKGIRTSWIMNEYRLPQHETERLQKAEISLCRVYKRAGVEDHPSLPRSLPTRASSSRGT
TSSSAKKSQEATNHASMERFQAFVGNPQQLDEKLSETSGSSCTDIGTSLGLSKHNTFMSLAPMTT
TLSQLCSTTLAPDCTTIFAGSSFVPTVNTTLDDLHRLVNFQQASMSQHQQQYHNNPNHPSQFSSL
QPQVQQSLALNMLPGPLQAAFTDRLWDWNSINEASKDFNNSPFK*
>Cc02_g36980
MGRGRVELKRIENKINRQVTFSKRRNGLLKKAYELSVLCDAEVALIIFSSRGKLYEFGSSGITKTL
ERYQRCSLSPQENAAERETQSWYQEVSKLKAKYESLQRAQRHLLGEDLGPLNVKELQNLEKQL
EGALLQARQRKTQLMIEQMEELRRKERQLGDLNKQLKIKVSLEMSSLEAAEGQGLRALPCLWSS
SVPSGSSMFPMHPSAMDCDPEPVLQIGYHHQYAPAEGPSAPRSMAIESNIIQGWAL*
>Cc06_g03390
MTTSSDPQSMPNSATPTATATNTDAYGCGSGKKIRKPYTITKSRESWTEEEHDKFLEALQLFDRD
WKKIEDFVGSKTVIQIRSHAQKYFLKVQKNGTIAHVPPPRPKRKAAHPYPQKAPKNVLVPVQASI
VYPSSVNPLAPGYPTWDDASVIASAQSSGMLPSQDEYTDIGSKGATRITNSYITGVGSSSKTLPAS
ELPKQGKQGSLLHGIPDFAEVYSFIGSVFDPTAEGHVQKLKEMDPINFETVLLLMRNLTINLSSPD
FEPVRTVLSSYDVNTKCMGVAAGNLVKNPAYLPC*
>Cc06_g01240
MESHQHHHHHQYGSSSGVHIDSGGGGGGDRFPQWSMQETRDFLMIRAELDPTFMETKRNKLL
WEVIATKMREKGYNRSAEQCKCKWKNLVTRYKGCETMEPEGVRQQFPFYNDLQTIFAARMQR
MLWMEAEGGAGSSKKKAAQLSSDDEDDNDDSEVERAHKKKRRKLKAGGGGGGGGASSASASA
AAGGNAVNSLKEILDEFMKQQMQIEMQWMKAYEAREEERRKRETEWRQTMEALENERLMMD
KRWREREEQRRIREEARAEKRDALITALLNKLQREDR*
>Cc06_g05680
MVVTDSEMTSHGSKVESPPLQSEQQQPKNHAFASLGSQSSIYSLTLDEFQNTVCESGKNFGSMN
MDEFLNSIWTAEENQAQVTSAAMVVAAANTNPNPKQLSQLGEANDAPIDQKGIITKQLSLPRQG
SLTLPGPLSRKTVEEVWTEIHQSQQEHEPPSNYTNPQNTGSSQQRQITFGEMTLEDFLVRAGVVR

EQTQPPPSPLPQQPQQPYGGTFYQNNDTNVMGTGFVTRPVIGGSANVVGYQPMPQTGDASAAY
PGSMKRGGGYAAQPTAACFGGRMGNGGSGGGYGQVQGLGMGSPVSPVSSEGLCANQMDGAN
PYGMDVGGMRGSGAGGGRKRIIDGPIEKVVERRQRRMIKNRESAARSRARKQAYTVELEAELN
QLKEENGHLKQALAELERKRRQQQVEESKMKAQTRTQRNDDKLMRTMRRTTSSPF*
>Cc10_g04070
MNMDELLKNIWSAEEFQTMEFTVGAKDTTGVGITTIPQRQQTLGEMTLEDFLLRAGVVREDAQL
AGKPNITGLLGDLSRTANNQASFGFGYQQPERNTGLVSGRVLESGNQMAMESANLPLNVNGVR
STQQQLGATQTQQTQLLPQQQHHPLFPKQPGLPYATPIAIPNSAQLGSPGVRGGIVGLSDPATSAS
LVQNGALQGVGVGMVNLGAGAVTIAAGSPAVSSDGLAKSNGDTSSVSPVPYVFNGGLRGRKCS
ALEKVVERRQRRMIKNRESAARSRARKQAYTMELEAEVAKLKEENQELQKKQAEIMEMQKNQ
VMEMMNQQRGGKRCCLRRTQTGPW*
>Cc08_g12100
MTRRCSHCSQNGHNSRTCPNRGVKLFGVRLTDGLIRKSASMGNLSHYSSSGSGSGSATPQNGLA
AHDSPGDTPDHPSAAAAAADGYASEDFVAGSSSSRERKKGVPWTEEEHRMFLLGLQKLGKGD
WRGIARNYVISRTPTQVASHAQKYFIRQSNVSRRKRRSSLFDIVPDESADATMVSRDFFSVNPPEA
ETQSNNELPAAAMEEEVESVDSANSIDVEAVPPKPDSSQYSYPVVYPTYVAPFFPVAFPMWSGY
GTEPTRQESHEVVKPTAVHSKSPINVDELVGMSKLSLGDSLGDGRPPLSLKLADGSRQSAFHANP
SSGTSGMNSSHSPIHAV*
>Cc06_g23190
MEIHFQQPEQEFAIPVSKMSNKFKGRSRSSGQNNKYVGVRQRPSGRWVAEIKDTTQKIRMWLGT
FETAEEAARAYDEAACLLRGSNTRTNFVPQQISPDSPLASRIKTLLNKRKNAKVPRSNTDMHESS
GATPSPANSVSVNNCIASSSNVISFEQKTPDGHLFDDAYKPDLSNCCAKGFEMEMGPSYHDQTHI
PSSGLQAQGFDHRFAFSTTQDHHQLLQLPKLNESGLPEIANHHMELSEFERMKVERQISASLYAM
NGVQEYMETVLDPIEALWDLPPLCSLLC*
>Cc10_g01980
MDDSGHRENGRHKPPQGQWLMQHQPSMKQIMAIMAERDAAIQERNLALSEKKAALAERDMAI
LQRDSAIAERNNAIMERDNAIATLQYRENSINSGNMSPCPPGCQITRGVKHMHHPQQHVHHQPQ
VNEPPYGSRDMPISDAIPISPGVLEPAKSRRTKRTKDPKAVTSTKKASKSSKKVKREGEDLNKTM
FGKSHEWKPGQEVGSGTDDLNRQLGVSKPDWKDQDLGLNQVAFDESTMPVPVCSCTGVLRPC
YKWGNGGWQSSCCTTNLSMYPLPAVPNKRHARIGGRKMSGSAFNKLLSRLAAEGHDLSNPVDL
KEHWAKHGTNRYITIK*
>Cc05_g10900
MTSGTRLPTWKERENNKRRERRRRAIAANIFAGLRMYGNYKLPKHCDNNEVLKALCNEAGWT
VEPDGTTYRKGCRPVDRMDIMGGSTSASPCSSYHPSPGTSYNPSPASSSFPSPASSPYAANINGDG
NSLIPWLKNLSSSSSSTSSTKFQLLCHGGSISAPVTPPLSSPTARTPRLKTDWDDSSARASWGSHSS
FLPLSTPPSPGRQAPPDSEWFAGMQIPQGGPTSPTFSLVSANPFAFKFESLRHTGSHMCTPGQSGT
CSPAIPAGSDVPMAEVISDEFAFRSIKGGLVKPWEGERIHEDCGSDDLELTLGSSKTRRANKRAVL
*
>Cc11_g03360
MATNRFVCEVCNKGFQREQNLQLHRRGHNLPWKLKQKTTKEVKRKVYLCPEPTCVHHHPSRA
LGDLTGIKKHYSRKHGEKKYKCNKCAKKYAVHSDWKAHTKTCGTREYKCDCGTLFSRRDSFIT
HRAFCDALAQESARNPPPLGIGASQLFGSSSNSSTMSMDMSQIGSQIARSTGVTGQFDNLIGSSMG
SALRPSKSMASSSYFLPESDQEDYHLENHSQHEFFSNKTLHHGLMQFSDLQNNSSASAGSSFFNL
SIFPSSSNAGSTLFSDHLVRQHLSSASIPSHYSTSIQSNNISPYMSATALLQKAAQLSSTTSNSTGAF
LKSFTSGASSSGTKPDPDPISSTQFGGIFGDYNTGNHIYEFMSSITGPRFGGADQGLTRDFLGVGPA
GELVRSMSGGGFGQREHGVEIKPLDSERKTAPESPPFGGGVNFR*
>Cc07_g12820
MVIGNKMPLDMEVSSVRSIQLTGQFRPGDDDYAQKVRKPYTITKQRERWTEDEHKKFLEALKL
YGRAWRRIEEHVGTKTAVQIRSHAQKFFSKVVRESNNGDSGSVKPIEIPPPRPKRKPLHPYPRKLS
ASVKSGALALEKQSAAANICSPAEANQSPTSVLSAFGSDAPGVTDSSTPEGSLSPVSSVIGGSSGF
VLSEPPNLSPPKPNASPSSSQVNNCSNQDEKLPLKLELFPEDNCFVKEGSEEVSSTQCLKLFGKTV
MVTDSCRPSSPTHLTCKMQPPSHSDGKFAQALPWNFVPIKYSQGDLERSWGALPFGTHGTLFCL
PLGGERSKPSETTPPSSLQWWTYQGGASFPFVRIHSPIPIKASCFGDQRDVQDMEIQKDGSSSDSN
AECECAEAESSRNLEAQSCQFLFAKEERAQASSRPCETSFLELKAGSGKCTKGFVPYKRCLAERD
ARSVVNSEEREEQRIRLCL*
>Cc04_g16510
MKEIMEMKDPEIKLFGKKIALLENGKRILVVVPAAGEDSSSVSGGENSVGSDSDLSMDSRKAEDE
KDQEKRETASDKDCPSGKLCSSEPRVKDPIAEELPILKSSSDSDGNSNNYSTDEDSPVKQPPKAEN
DQSDATDSQQKTLKKPDKILPCPRCNSMDTKFCYYNNYNINQPRHFCKSCQRYWTAGGTMRNV
PVGAGRRKNKNSASHCRHITISEALQAARIDTPNGFHHLNFKPNGTVLSFGSDSPLCDSMASVLN

LAEKKSPNGSQNGFYKLDHGISVSLKGTENGDDCSSGSSVTTSNSTVDGAKNGLQEPVMQQING
FPSPVSCLPGVPWAFPWSSTVPLPAICPPGFPMPFYPTPFWNCGVPGAWSIPWLPPVFPTGNQKNS
GSGPNSPTLGKHSRNGELIKQNNPEGKDSQEQRASEGSILIPKTLRIDDPDEAAKSSIWTTLGINYD
SISRGGLFKALQPKGDEKKHLTTASPVLQANPAALSRSVTFQESA*
>Cc08_g03540
MGSSEMDKSSKEAKEAKESKTPPQEQPSAPSTGTPDWSGFQAYSPMPPHGFLASSPQAHPYMWG
VQHLMPPYGTPPHPYVAMYPPGGIYAHPSIPPGSYPFSPFAMASPNGVAEASGNTPGNTEVDGKP
SEGKEKLPIKRSKGSLGSLNMITGRNNEPGKTAGASANGAYSKSGESASDGSSEGSDANSQNESQ
MKSGQRQDSAETSQNGSAAHGSQNGGPNTPHSMVNSVPMLPISAPGAPGGLSGPTTNLNIGMDY
WGSAASPTVPAMRGKVPTAPVAGGMVTGGRDGVQSQLWIQDERELKRQRRKQSNRESARRSR
LRKQAECDELAQRADALKEENASLRAEVNRIRSEYDQLLAQNASLKERLGEVPGPDDPRSSRNE
QHAGNDAQHSGQAEPLQRGQ*
>Cc02_g27930
MNYMLTNGQGCWSDIARNAGLQRCGKSCRLRWINYLRPDLKRGAFSPQEEELIVHLHSILGNR
WSQIAARLPGRTDNEIKNFWNSTIKKRLKNNNAITSTSSPNTSDSSDPRAIMLGGLMQPMQAEQD
VFSICVDSSSSSPPCMQAILSITQGNPFNPFPPLHDSAAYLDINGSASAAGAAGLYNLSAAHVGGG
GGGSAGSLGDYGLVEPYVMGLESDLSLPALEGRAFDNINAANDQSVLDKRLVHNNHHFNGNEN
IKVVDDFVGIGNHWNGENLRMGELDWEGLLANVSSLPYLDFQVE*
>Cc05_g08380
MSDEDDAGLPPTSTNTKPTTPTSPSPSPPPPPSSSADIFPPHPPPRPSTTTKATSFPIREDCWSEDATH
TLIEAWGSHYLDLNRGNLRQKHWQEVADAVNADHAHTKKLHRTDIQCKNRIDTLKKKYKLEK
AKFLQSNGRVVSTWPFFASLDSLIGDSFIKANAPPSGTPPFPVLPPLPSAVPVGPRSKRPAPAQAM
GEEAVFRRNFSAMAAAAAAVAEDDEDEEESDTSSAAAVGLGSGGMRRKKRRGAAGKVAAEGY
RKLAEAIRGFADIYERVEEAKQRQMVELEKQRMQFAKDLEIQRMKLFMESQVQLEKLKRSKRN
SQSGDGYL*
>Cc06_g02740
MVFSSIPAYLDPANWQQPSNHQAGNSSGNHPPLQPPAQQQPVPSFPPAQPHAGGGPGAVSIRPGS
MAERARLANIPLPEAALKCPRCESSNTKFCYFNNYSLTQPRHFCKTCRRYWTRGGALRNVPVGG
GCRRNKRTKATSSKSPVSSADRQTTSSSTGTVSSNSTPSANVLGLTPQIPPMRFLSPLSQLSDHYSP
AGDISLNYGANNSAPLLGTSEMNFHIYPNNLLSCGLGGGAASLLPGGGIEQWRLQQAQQFPFLG
GLEASPPGLYSQLQASSVEPSGFVGETSRHHVRPKLSSSILTQQASVKMEDNHQELNLSTQLMGIP
GNHDQWNGTAAAWTTDLASFSSSSTTNPL*
>Cc04_g11850
MASSSSYNSPCAACKFLRRKCMPGCIFAPYFPPEEPQKFANVHKIFGASNVSKLLNELLPHQRED
AVNSLAYEAEARVKDPVYGCVGAISFLQRQVERLQKELDAANADLIRYACNDHIQPELSAPHGA
MHQVHQPMTPRQRPVEYNNTRRMGNEGGGFYQTPNFQYPYHLPWNDNDIHRYGGGGGGGGG
GHI*
>Cc07_g01000
MSETNHNNSPTPPPPAEIKDFRICIADKQENTTTSAKKPHQLAPKRSSNKDRHTKVEGRGRRIRMP
ALCAARIFQLTRELGHKSDGETIQWLLQQAEPSIIAATGTGTIPASALAAAAAGAGGSVSMSAGL
HPPKISAELGAHHPPHMDIAGSGQGAGSTGASRTNWPMVGGSLLRAPHMGMPTTTAGIWPPTSA
SGAVSGFGFQSSSSPAPAATSLGTESSNYLHKLGFPGFDLPAATNNLGPMSFTSILGAATDQQQQL
PGLELGLSQDGHVGVLNPQTLSQIYQHMGQARAHQQQQHQDQQQQQPSPNDNSQGSGQ*
>Cc00_g06830
MQPRDSPHNGVPVSPSNSGEVVSCVVLKIESGSLQQKQISASESCEPEAHKVPPLVKNEKDELNQ
WQSHGAGNHSTAYGETEVLDSGKDVAISNQVSIVPKKELVSDGQLHASVSSKQLIPENGNRASQ
FNQVSVQPKEELGRSEQNLSPHTGSNTPMSGEKLIPESGTHVSQFNHVSIITKMDPDGPEQEQGTD
AGKDASLPCEGGGTDSSVLEKSLQHMQNTNMRVCTSSSDQEKITYSAKPEKVLYKLQPRRNPDS
GVHATQSEQGSNSPRIREKALDDGYNWRKYGQKLVKGNVFVRSYYKCTYSTCRAKKQVERLH
DGRLTDIKYIGKHEHPKLQSSPQCTAFVSPSEVSKADMPAIATSEAEDELVVAHNDKPQPIDPAET
PRELAATASNNSTGKAVPQLHNPRDDIDNDISPTSKRQKRETCDVHNNQVKKTHCESRQVVHM
MSEVDIVNDGYRWRKYGQKLVKGNPNPRSYYRCSNAGCTVKKHVERSSHDPKVVITTYEGKH
DHDMPASRTVGHSATESGTNGTTMKGEAKSEIGEHNAVGMDLIVKIGAN*
>Cc10_g02160
MARGKIQIKRIENQTNRQVTYSKRRNGLFKKAHELTVLCDARVSIIMVSSTQKLHEYISPTATTK
QLVDQYQKAVGVDLWSSHHEKMQEQLKKLKEVNRNLRKEIRQRMGESLNDLSYDELGFLIEDV
DNSLRAIRERKYKVIGNQIETHKKKVRNVEEIHRNLLLELDARGEDPHYGLVDNGGGDYNPVLG
YPRVLALRFQPTQPNLHSGGGSSDLTTFALLE*
>Cc05_g00300

MNPCFPDWNFGVEFPEPILKNKPLGMDNELVELLWENGQIVLHPQSHHHHHPKPGTADQQENQ
SRQVDKHNHDQSVSRGSTGGGSCQNQVTSLIQDTETVSWIDDPFDKEFTSDFLSEFPISNPVEQGP
HEDDKFKKFGISQDLHNHPVQLPNDKPSDVINSLPPGFHNFDSAQPNHSHLPRAANAPLSAKADL
RSSSDGVSNRTLGGEAREYSSAKTVGTSHCGSNLVVNDTDTSRVSSGGIANHRGFSGAMAKDHR
VGKMSSQSDGLQTDQTEETAITSSSSDGSETSFGRTCNQSTGTNSHKRKSRDAEDSECQSKAAEL
ESAARKKPAVKSGSSRKSRAAEVHNLSERRRRDRINEKMRALQELLPKSNKTDKASMLDEAIEY
MKSLQLQLQMMWMGSGMAPMMFPGVQHYMPRLGMGIGPLAMPSIHSQMHLPRLPLLDQATIP
NQAALCHQTTMFNPMNYHAQMQNSKLSEQYANYMAFHPLQNASQQLNVFGFGSNTAQQHNH
SLAPSGNSNGPSVG*
>Cc10_g07810
MGRAPCCDKANVKKGPWSPEEDAKLKSYIEQHGTGGNWIALPQKIGLKRCGKSCRLRWLNYLR
PNIKHGGFSEEEDNIICSLYISIGSRWSIIAAQLPGRTDNDIKNYWNTRLKKKLLGKQRKDQHARK
STSQKQEMLRKGMRESINHMVSSSSSDYNSNQSPYWPELPVLPPVPYSNEEPRFNDHASIRKLLIK
LGGRFSDDDDQPTNGTMNTNLQYPLDSTNSLVQVQPLYDQQINMLSSAPLDVLNTTSSLPETLY
TIDAADLSTLQGQNSFQAGLEQMICNNPQRLDGLEFLCGDILINHRTGNTCGGSSDWGEMNSLV
LPAVASGYEGLQQGTLQECAIDQLRYLGP*
>Cc02_g24540
MLDYEWGNPSAVMFTGDDSTQDADQNRQLFDPYGTQNFGEATAALVHQNAHYSAAAHHQHT
GNPFHHPHDPQGQSSNAHFSSLFDPRAAYGASSFNPHHQASMLSLEPAGSTGFMVIPKSEPVVGG
ADFTAAGRIGLNLGGRTYFSSSEDDFVSRLYRRSRVVEPGSVNSPRCQAEGCNADLTNAKHYHR
RHKVCEFHSKAATVIASGLTQRFCQQCSRFHLLSEFDNGKRSCRKRLADHNRRRRKSQQQQQP
NQEHCNTNSKKSPNDNTRSPPDSGAHSSSVTVAVSPPRISLDCFRQRASYQGGGAAATNSSASTS
SLFFSDG*
>Cc05_g16190
MEMETMSSVVTTNSKDEDDDVPLPGFRFHPTDEELVGFYLRRKVEKKPISLELIKQIDIYKYDPW
DLPKASTVGDKEWYFFCKRGRKYRNSIRPNRVTGSGFWKATGIDRPIYSAGGEGRDCIGLKKSL
VYYRGSAGKGTKTDWMMHEFRLPAEHDNIKSTKHIDAKNIAQEAEVWTLCRIFKRNVSYRKCM
PDWKEISGKRLSNAVDASSKTCSMEFDNNDKKPLINNNNNNNNNSVHGLGHVDITNQLLMSQ
LSSFSQAPSSTTASYASFASSDATDFLKHGDWDELRSMVEFGAGPTFF*
>Cc02_g37000
MISFFFKGGSFSLHNCYPFYDGIYQKKALRINQSITPTLLSFSRYLVFLDFWLSPHSSLSHATIFLLK
EEKGVGKEKQVQVRLEEFGFFPFVGISPLCLQPKGSLGFCQFCVKMVRGKTQMRRIENATSRQV
TFSKRRNGLLKKAFELSVLCDAEVALIIFSPRGKLYEFGSSSMKEIIERYQKHAKDVRANNPSAEQ
NMQQLKQETASMVKKIELLEASKRKLLGEGLVSCTVEELQQLERQLERSVNCIRARKMQVFQEQ
IEKLKEKEKVLEAENDKLLEKCGAEPPQTSKENTEIVPCTESSEVSDVETGLFIGPPERRNKLVLK
N*
>Cc11_g13310
MTCTAAMAFFPANFMLQTSHHDQDHHDSQQPPTSLSTPILSSCAPQDFHGVASLLGKRSMSFSG
VDVCDQENHGEDDLSDDGSQMGEKKRRLNMEQVKTLEKNFELGNKLEPERKMQLARALGLQP
RQIAIWFQNRRARWKTKQLEKDYEVLKRQFEAIKAENDALQAQNQKLHAEISALKNREPTESIN
LNKETEGSCSNRSENSSDIKLDISRTPAIDRDSPLSTHPTASRSLFPTSIRSAGGMTTTQLFHNSTRP
DLQCQKMDHSTVKEESLCNMFGIDDQAGFWPWLEQQHFN*
>Cc02_g39990
MDTYSSGEELIIKARKPYTITKQRERWTEEEHNRFLEALKLYGRAWQRIEEHIGTKTAVQIRSHA
QKFFTKLEKEALAKGTPIGQALDIEIPPPRPKRKPCNPYPRKMSIGLPTSQVESKDGKLGNPSSSLP
QAEHILDLENEPAAEKPEGEKFEDAKENRDEGESNKCFNLLQASPGIPPSSANTCSSTMASGKSSV
LMEFVPIFNEVVNRDETTESYVTVEAGPNQNSDKFYTIQSFEENGTCNSRNWANAHSLNDKPAQ
NKGNVPEQSENIDTSPKNGVQSPKSQPRHVAVHILDGSLRMNAPNVSAHMPYEESLFLEIGGVNS
NIFSTLSSSATSEHQSSASRFSISEPFVGFNPILTPVHQEDYHSFLHASSTFSNLIVSALLQNPMAHA
AASFAASLWPCSTTEAPAESPGGGTGGFTARQMNAAPSITSIATATVAAATAWWTAHGLLPLCA
PFHMGLNYVPVSGSAVPAVTNQPRVDITERREEISGPPLDAKHLEPEYCEALQEQHSTSKSPTLSL
SDSVESKGLNSSNKSTAKKTDSAAVTDLNDSNKPKGRKRVDRSSCGSNTPSSSEVETDALEKLEK
GKEELREPVVSQPTADSSGRRSRSSSSTSDSWKEVSEEGRLAFQALFSREVLPQSFSPPHDSKNKG
KNTNEKNKDNADGKGDKGYLLDLNGKMCTNSATHQDLENNLSLRCLNITEGGLLTTGKLKMR
RTGFKPYKRCSVEAKENRLVSAGSQEEEKGTKRIRLEGEASTH*
>Cc02_g39490
MEKGVFVRFKKKMSGKTRCKHKYRAAMDFMYWLHAQLAKLWFNCWLIMEMLLQQPPAPPCI
NEDDLAPSSSGSTTTTTTPTAASSSRSDTSSSSAARGGTRHPVFRGVRKRRWGKWVSEIREPRKK
SRIWLGSFPSPEMAARAYDVAAYCLKGPKAQLNFPDDIQLLPRPLTTTARDIQAAASLAARVAM

AEGKKTATIGSDDFWRDIELPELIEAWPGENDNSTSVAVRRDYVWELRYWSASSSLSSCSGMFS
GDIATSPME*
>Cc11_g17110
MGRGRVELKRIENKINRQVTFAKRRNGLLKKAYELSVLCDAEVALIIFSNRGKLYEFCSSSSMLK
TLERYQKCNYGAPEPNISTREALELSSQQEYLKLKARYEALQRSQRNLLGEDLGPLNSKELESLE
RQLDMSLKQIRSTRTQVMLDQLTDLQRKEHALNEANKTLKQRLMEGNQVNLQWNPNAQDVG
YGRQPAHAQGDGFFHPLDCEPTLQIGYQNDPITVAAAGPSVNNYMAGWLP*
>Cc07_g10660
MEPQQQQNHQQQQQQNQLLPNEDGGCSKNGFLCRQSSTRWTPTTEQIRILKDLYYNNGVRSPT
ADQIQKISAKLRQYGKIEGKNVFYWFQNHKARERQKKRLTTDISAMQRGVWRSDYQESICSTKY
PNITSGAPSSSASVGGLHAAGQVGNYGYGSFAMEKSFRDCSISPSGKASGSMFQNFSWAGAGAG
AEPYPSPYQFLEKKTCFVETLDDQEQEEEPVPELETLPLFPMHGEDVSGFCTNKQPEQNCYYTDW
YQPNGNVGYSRTSLELSLNTYGGRSQNSP*
>Cc02_g11300
MNSGNEPMNEVEKSLDPQLWHACAGGMVQMSPVNSKVFYFPQGHAEHAHKSVDFGTFCRIPPL
ILCRVSSIKYLADTETDEVFAKIRLAPLRGNECSDDDGDDGLLAFDKNDGQEKPSSFAKTLTQSD
ANNGGGFSVPRYCAETIFPRLDYSAEPPVQTILAKDVHGEIWKFRHIYRGTPRRHLLTTGWSNFV
NQKKLVAGDSIVFLRAENGDLCVGIRRAKRGIGGEEENRIMRNPKGGTNNSDIGARGRGKVRAE
SVVEAANLAASGQAFEVIYYPRASTPEFVVKASAVKAAIRIQWCSGMRFKMPFETEDSSRISWFM
GTVSSVQVDDPIHWPNSPWRLLQVAWDEPDLLQNVKRVSPWLVELVSNMPAINLSPFSPPRKKL
RLPQPPEFPLVGQLPMPSLFSNPLSPSSPLCCLPDKIPAGIQGARHAQFGLPSSEPHFNKLQAGLFPF
KLKQLEHAAAAASRIPNSSCFMEDYESKDNVSCVLTIGNSVQGSKPNVRTEAPLFVLFGQPILTE
QQISQSSSGDTARSSLSERNPENTVTVSGGSGSGVLQSGHPENSLDQVLPWYKDPKLEFGLETGH
CKVFMESEDVGRTLDLSVFGSYEELYGKLAEMFGLERSEMLSNVLYQDPAGVVKHSGDEPFSDF
LKAARRITILTDSGSDNVGR*
>Cc10_g02460
MASSSGNSSGSTSSQIQNSGSEGDLQLLMDQRKRKRMQSNRESARRSRMRKQKHLDDLTAQVA
QIKEENSQILSTMNITTQQFLNVEAENSVLRAQLTELTQRLQSLNEILSYMNTSNGMLDALELQPN
SEIFMNNPWNLTYPNQPIMASADMFPY*
>Cc11_g15060
MALEQLSLAVAAAPPHSDSPSPSPALNGRQPSTTALDPHKDDDSAKVPRLPRWTRQEILVLIQGK
RVAENRVRRGRTAGLAFGSAHVEPKWASVSSYCKRHGVNRGPVQCRKRWSNLAGDFKKIKEW
ESQIREETDSFWLMRNDLRRERKLPGFFDREVYDILDGGGRADGATPAAEEGAEELVLALAPSA
AEGPEEADAETEAVFDSGRSAAADDGLFSDFEQSVQEEVVGTPDKEFRPANDTPVTAVPAPTPIS
EKQYQPISQGHPAQGMFRYLPTVTNLSNVFFSCSLEMPCSSSKTNFCLVFQDVYPIAVKMGEFLL
*
>Cc03_g07150
MDALHNPDVAGAVAGCFMLDDEDDLLNFSLDDGEEEKENKNPSPFLKDPYSFSPSNTLISHQDD
DLSRSPSFPEFEEELEWLSNKDAFPAVETCFDLLSETPDFDGLNHQSPVSVLENSSSSSNSNGSNSS
NGSAVMSCCENLKVPSSFPVRPRSQRRRRKRRSGFGDLPSQEWQWWNHVNIKSNRQELALPPVP
VKANTSATIGRRCLHCQADQTPQWRAGPMGPKTLCNACGVRYKSGRLVPEYRPASSPTFSAAL
HSNSHRKIVEMRRQKQPGIGGIMANGSCGYRVG*
>Cc07_g02340
MDSGRIYFDPSCHGNMLFLGSGNPVFPGARSVLNVEETLKRRPFLRSPEELFDEEYYEEQLAEKK
RRLTPEQVHLLEKSFEAENKLEPERKTQLAKKLGLQPRQVAVWFQNRRARWKTKQLERDYDQV
KSSYDSLRSDYDSVVKENEKLKTEVLSLTEKLQQGKEVVVEPRSKQKSDALPVDELLASDPQFN
VKVEDRLSTGSGGSAVVDEDGPQLVDSGDSYFPCDDYAAGCVTPAADGVQSEEDDGSDNGQN
YLSNVFVAAEQQNQEGEPVGWWVWS*
>Cc11_g07170
MVEEKENKRPVIFRDIRRYYCEYCGICRSKKSLISSHILSHHQEELKQKEEENGQAKDEPKMNTC
EHCGVSFRKPAYLRQHMQSHSFEYQVGGPPEKILQASLFHAVFSYERPFKCLVADCHASYRRKD
HLTRHLLQHQGKLFECPLDSCKCGFAYQGNMKRHVKEFHYDSSSNDVTSPKQYVCTEVGCGKV
FKFASKLRRHEESHVKLDTVEAFCAEPGCMKYFTNGECLKEHVRSCHQYVICETCGTRQLKKNF
KRHLCSHEAGCSQSKVKCSFEGCLLSFSNKSNLNQHVKAVHLGLQPFSCRFPSCGMTFSFKHVR
DNHEKSGRHVFTRGDFEESDEQFRSRPRGGRKRQYPVIETLMRKRIVPPCESDPILNEASDYLSRF
FSTESEDEL*
>Cc10_g03290
MMWEAGGSTASSSAAGGSGGGGGGGGEGGGSGGVVNGRRKPSWRERENNRRRERRRRAIAA
KIYAGLRAQGNYNLPKHCDNNEVLKALCAEAGWIVESDGTTYRKGCRPPPIEIGSTSANITPSSSR
NPSPPSSYFASPIPSYQPSPLSSSCPSPTRQEPNMASHPFAFLRNSIPSSLPPLRISNSAPVTPPLSSPT

RLPKQIFNLEALAKESMNALNIPFFAASAPASPTRGQRFTPATIPECDESDSSTIDSGQWMNFQTY
APNMVPTSPTFNLMKPMAQPVSPMDVVSGKGKGIEFDFEKLPVKAWEGERIHEVGLDDLELTLG
SGNARI*
>Cc06_g03810
MLVMSANMFTVPPSVNGFSPEPADTNPTSSNSNPKSNSTTTNPAGKKKRNLPGNPDPDAEVIALS
PKTLMATNRFICEICNKGFQREQNLQLHRRGHNLPWKLKQRTTKEQVKRKVYICPEKTCVHHDP
SRALGDLTGIKKHFSRKHGEKKWKCEKCSKKYAVQSDWKAHSKTCGTREYKCDCGTLFSRKDS
FITHRAFCDALAEESARITSVAANNFNFKTDQQHLNGGALLNHPGSFPHGLSGPSGISQIGVDFAT
GNQLKPRLSLWLDQASNPQLNHADMPPSQNLYMPSASHHDIMQMSSAADVFGNYGMPWQQLE
KSSAPPSSASSSSAANLSLSPMMPQQVLKDEGAGGSINTQTLSSLYSPSAASNNDISHSKSSATPM
SATALLQKAAQMGSSKSTAAIFGNGFGIMNPSSSSSSPAVAANMIAPAAFSSGLAQNRTEMMQV
FGKQTDNMMGGGSNMTSSTSSTLTNNLDHQPMVQTSTVADSQTVASLGGFHRHPGFNAVVEH
ASLTRDFLGMGGDGGRPFLPHELAKFASMSSAMGLGHFSSNH*
>Cc06_g17200
MGRPPCCDKVGIKKGPWTPEEDIILVSYIQEHGPGNWRSVPTNTGLLRCSKSCRLRWTNYLRPGI
KRGNFTPHEEGMIIHLQALLGNKWAAIASYLPQRTDNDIKNYWNTHLKKKIKRFQAGSDPQILSD
SSTATINQLVSRAFCDDGKHNNTSAALKLNQNTTVYASSAENISRLLEGWMKSSPRSNNITNSPT
GGDKTRATGDSVASISGSETSGNEEKIIMSSDHHAHEQKPKLENNPPLSFLENWLLDEAAAQVEG
VMEIPSIF*
