# Supplementary material for: Genome-wide analysis, transcription factor network approach and gene expression profile of GH3 genes over early somatic embryogenesis in Coffea spp
Source: BMC Genomics. 2019 Nov 6;20:812. doi: 10.1186/s12864-019-6176-1 (PMC6836404; doi:10.1186/s12864-019-6176-1)
Supplement: Supplementary file 3 — Additional file 3: Figure S1. Gene distribution of CcGH3 members on C. canephora chromosomes using the option “locus search” in the Coffe Genome Hub website. The colors within each chromosome indicate ancestral blocks corresponding to the 7 core eudicot chromosomes (DENOUED et al., 2014). Red lines stand for the position of each CcGH3 over chromosomes. The most CcGH3 members are not presented in any chromosome, so they were allocated in chromosome 0 (not available). The genes are distributed as follow into each chromosome (chr): chr1: CcGH3.9; chr2: CcGH3.10, CcGH3.11 and CcGH3.12; chr5: CcGH3.13, CcGH3.14 and CcGH3.15; chr7: CcGH3.16; chr10: CcGH3.17. [file 12864_2019_6176_MOESM3_ESM.pdf]

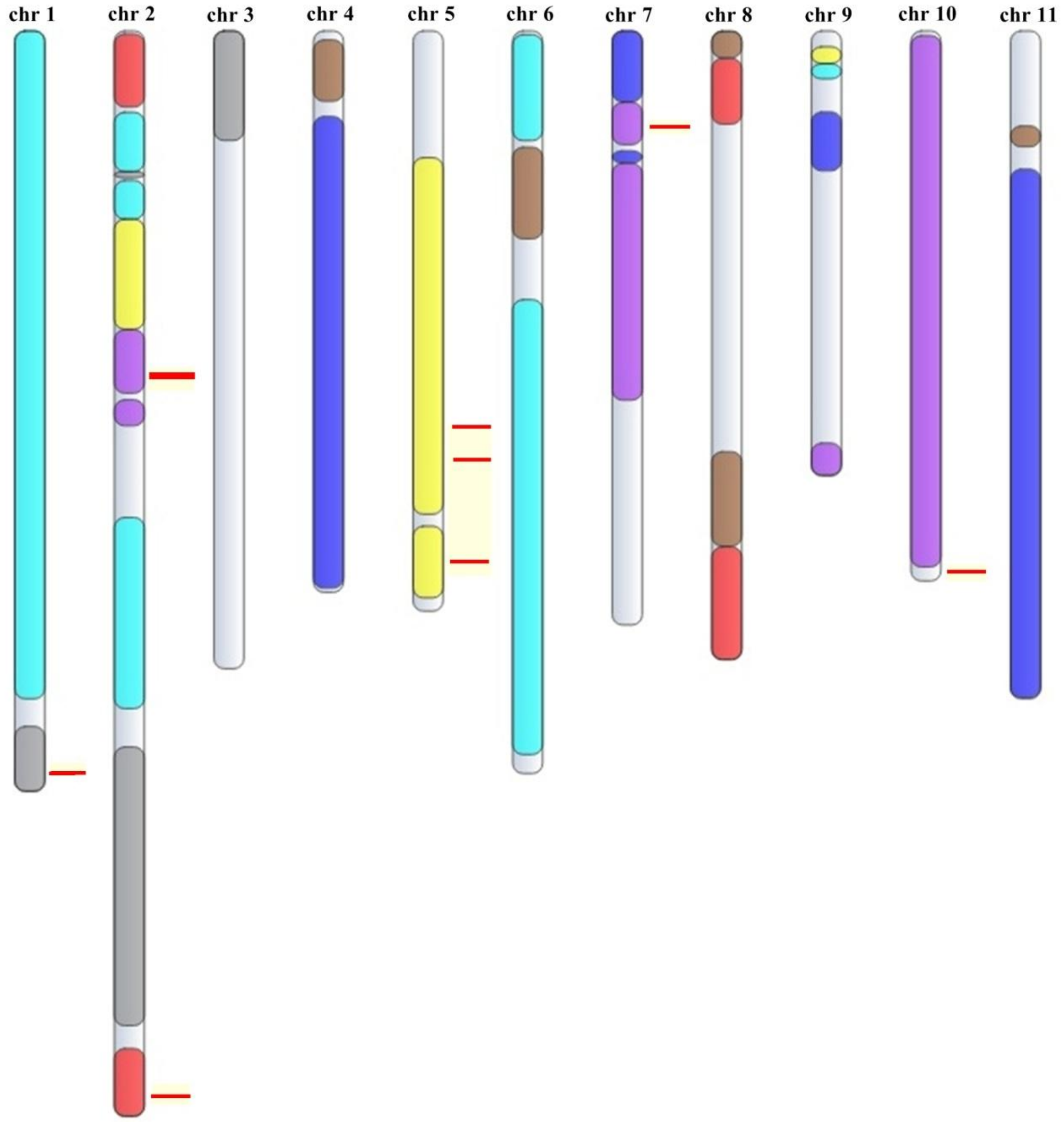

Supplementary Figure S1. Gene distribution of *CcGH3* members on *C. canephora* chromosomes using the option "locus search" in the Coffe Genome Hub website. The colors within each chromosome indicate ancestral blocks corresponding to the 7 core eudicot chromosomes (DENOUED et al., 2014). Red lines stand for the position of each *CcGH3* over chromosomes. The most *CcGH3* members are not presented in any chromosome, so they were allocated in chromosome 0 (not available). The genes are distributed as follow into each chromosome (chr): chr1: *CcGH3.9*; chr2: *CcGH3.10*, *CcGH3.11* and *CcGH3.12*; chr5: *CcGH3.13*, *CcGH3.14* and *CcGH3.15*; chr7: *CcGH3.16*; chr10: *CcGH3.17*.
