# Supplementary material for: Genome-wide analysis, transcription factor network approach and gene expression profile of GH3 genes over early somatic embryogenesis in Coffea spp
Source: BMC Genomics. 2019 Nov 6;20:812. doi: 10.1186/s12864-019-6176-1 (PMC6836404; doi:10.1186/s12864-019-6176-1)
Supplement: Supplementary file 4 — Additional file 4: Figure S2 Tridimensional structure model for the four GH3 proteins selected in Coffea canephora. [file 12864_2019_6176_MOESM4_ESM.pdf]

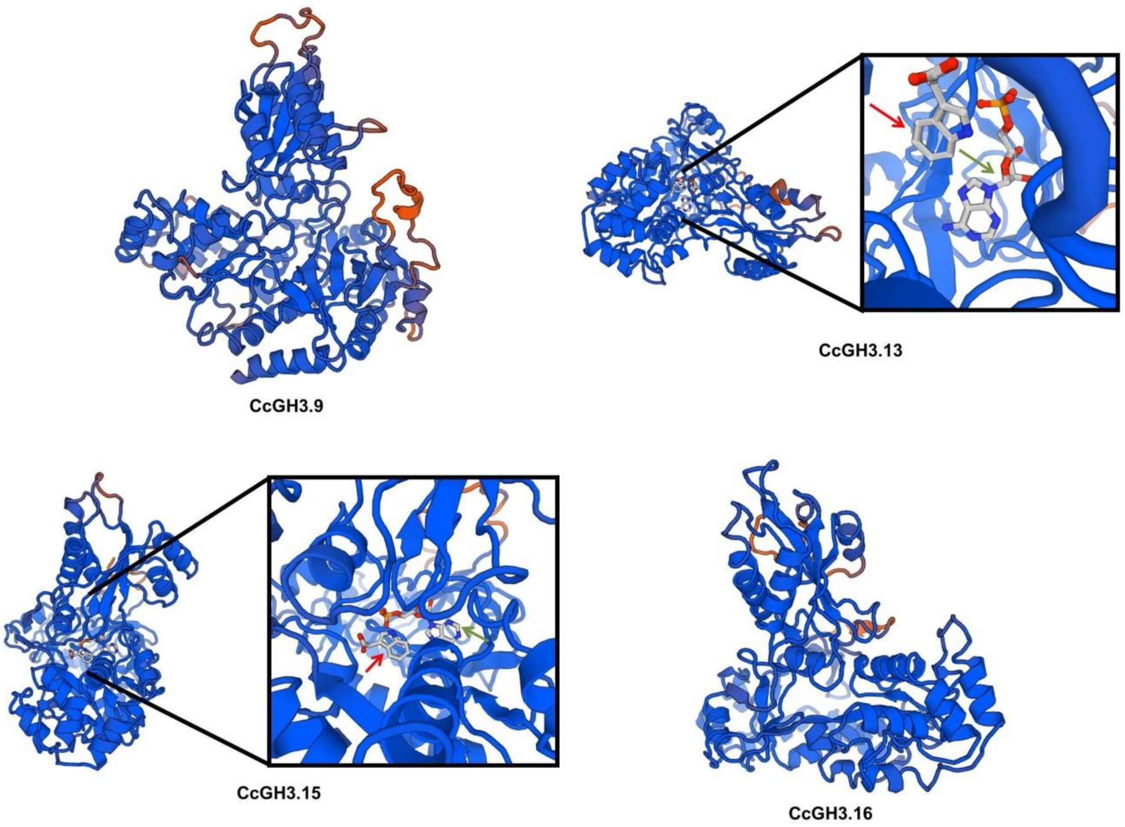

Supplementary Figure S2. Tridimensional structure model for the four GH3 proteins selected in *Coffea canephora*.
