# Supplementary material for: Genome-wide analysis, transcription factor network approach and gene expression profile of GH3 genes over early somatic embryogenesis in Coffea spp
Source: BMC Genomics. 2019 Nov 6;20:812. doi: 10.1186/s12864-019-6176-1 (PMC6836404; doi:10.1186/s12864-019-6176-1)
Supplement: Supplementary file 5 — Additional file 5: Figure S3 RNA integrity assessment by agarose gel electrophoresis. This figure was cropped from the original picture to exhibit the RNAs extracted from three biological replicates referred to each cell type used for RT-qPCR analysis. The bands clearly separated and visible represent two rRNA subunits point to the good RNA quality. From the left to the right are the samples: Embryogenic cell suspension, biological replicates 1, 2 and 3; Embryogenic calli, biological replicates 1, 2 and 3; Non-embryogenic calli, biological replicates 1, 2 and 3. [file 12864_2019_6176_MOESM5_ESM.pdf]

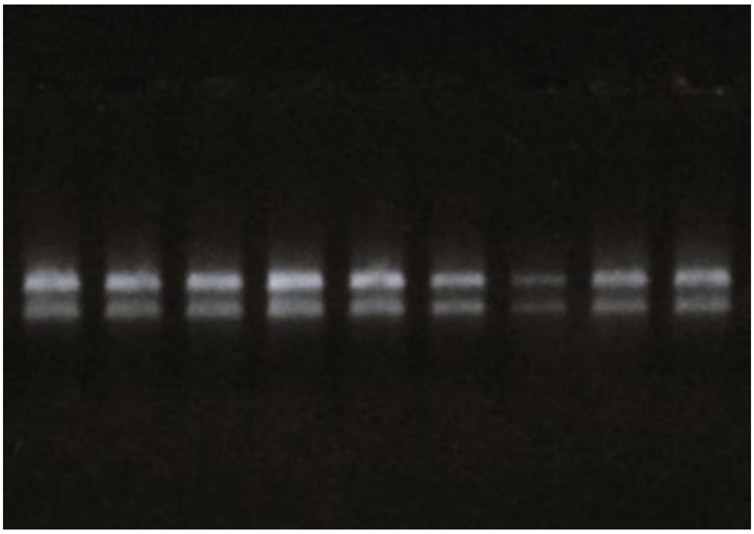

Supplementary Figure S3. RNA integrity assessment by agarose gel electrophoresis. This figure was cropped from the original picture to exhibit the RNAs extracted from three biological replicates referred to each cell type used for RT-qPCR analysis. The bands clearly separated and visible represent two rRNA subunits point to the good RNA quality. From the left to the right are the samples: Embryogenic cell suspension, biological replicates 1, 2 and 3; Embryogenic calli, biological replicates 1, 2 and 3; Non-embryogenic calli, biological replicates 1, 2 and 3.
